# Automated Segmentation and Analysis of Histopathological Breast Cancer Images for Enhanced IDC Diagnosis and Assessment Using MobileNetV2+U‐Net With Label Propagation

**DOI:** 10.1155/ijbc/5948413

**Published:** 2026-01-31

**Authors:** Vijaylaxmi Inamdar, S. G. Shaila

**Affiliations:** ^1^ Dayananda Sagar Academy of Technology and Management, Bengaluru, Karnataka, India, dayanandasagar.edu; ^2^ School of Engineering, Dayananda Sagar University, Bengaluru, Karnataka, India

**Keywords:** breast cancer, diagnosis, histopathological images, invasive ductal carcinoma, label propagation, MobileNetV2, segmentation, U-Net

## Abstract

Breast cancer remains the most common cancer type among women, with invasive ductal carcinoma (IDC) responsible for almost 80% of cases. The exact histopathological segmentation of IDC is the premise of diagnosis, but manual observation of hematoxylin and eosin (H&E) stained slides is very time‐consuming and results in interobserver variability. This work presents an automated IDC segmentation method with a lightweight hybrid deep learning framework by integrating U‐Net with a MobileNetV2 encoder and a label propagation refinement module. This hybrid model leverages MobileNetV2′s efficient depth‐wise‐separable convolutions for feature extraction, U‐Net′s encoder–decoder precision for boundary localization, and the label propagation step enhances spatial smoothness and anatomical consistency. Experiments are conducted on the BACH 2018 and BreakHis datasets at multiple magnification levels (40×, 100×, and 200×). The model reaches a precision of 94.85%, Dice coefficient of 94.63%, F1‐score of 94.56%, and AUC of 94.65% on the BACH dataset and a precision of 93.87%, Dice of 94.24%, F1‐score of 94.18%, and AUC of 93.93% on the BreakHis dataset. The proposed model surpasses several state‐of‐the‐art techniques such as CNN and transformer‐based models, including DeepLabV3, Mask R‐CNN, Swin‐UNet, and ViT‐Histo. Cross‐dataset validation yields a Dice of 92.10% and AUC of 93.70% from BACH → BreakHis, confirming robustness under domain shifts. Explainable AI analyses using Grad‐CAM and SHAP confirmed accurate localization of diagnostically relevant regions. The proposed hybrid model of MobileNetV2 + U‐Net with label propagation presents a computationally efficient and clinically reliable solution toward real‐time, AI‐assisted breast cancer histopathology.

## 1. Introduction

Breast cancer is considered one of the most common and deadly cancers, occurring in millions of women every year. The current global survey as per GLOBOCAN 2024 was recently published by the International Agency for Research on Cancer (IARC) and the American Cancer Society (ACS) [1, 2]. In the year 2022, there were nearly 20 million new cases resulting in an estimated 9.3 million deaths from breast cancer, which represented 11.6% of all new cancer cases and 7% of all cancer deaths globally. These statistics represent a sustained and increasing global health burden, especially in low and middle‐income countries where late diagnosis and access to advanced screening technologies continue to present significant hurdles. The survival rate improves when doctors diagnose breast cancer at the right time and start treatment right away. The most prevalent form of breast cancer exists as invasive ductal carcinoma (IDC), which pathologists identify through histopathological examination. The method requires tissue collection for microscopic examination to detect malignant cells. The accepted gold standard for breast cancer diagnosis through histopathology faces multiple obstacles that affect both diagnostic precision and operational effectiveness. The manual assessment method used in histopathological diagnosis creates the biggest challenge for achieving accurate results. Pathologists use microscopic examination to detect small tissue abnormalities by searching for abnormal cell structures, nuclear irregularities, increased cell numbers, and abnormal cell divisions [3]. These features are hard to detect and show significant variability between cases, patients, and within the same sample [4]. Manual review is time‐consuming and has inherent human variability, especially when determining borderline cases or when first identifying malignant changes. Variability can be exacerbated by factors such as fatigue, differences in skill, and case load, which can exacerbate timing and variability issues. Therefore, to address these problems, attempts have been made for automated approaches with artificial intelligence (AI) and deep learning (DL) techniques. Nowadays, AI tools support and augment human tasks in detecting and identifying disease. Various approaches, including DL, have been successful for classifying medical images, including X‐rays, MRIs, and histopathology slides. However, beyond image classification, precise localization and segmentation of tumor areas is considered an important aspect for breast cancer diagnosis, which determines tumor margins, malignancy grade, and monitoring through treatment response. In breast cancer, the timely detection and the advent of AI assistants that quickly indicate suspicious regions for actionable treatment and avoiding of the development of tumors [5] is very crucial for survival. Thus, there is a need for close collaboration between AI researchers and practicing pathologists wherein AI researchers optimize the algorithms based on real‐time problems and pathologists ensure the integration of these algorithms into the clinical aspect of hospitals. Still, numerous challenges exist, such as model interpretability, adaptability to different staining protocols, and the financial implications in resource‐limited clinical environments. Though these challenges are gradually addressed, the vision with respect to making sure that the technology is available timely to every patient irrespective of location or resources with accurate cancer detection always remains challenging. Thus, the goal of our work is to develop an automated segmentation framework for analyzing and identifying IDC in histopathological images, assisting pathologists in early diagnosis and clinical assessment.

Objectives specific to this study include the following:

1. The work proposes a lightweight hybrid U‐Net model integrated with a MobileNetV2 backbone that incorporates spatial precision and computational efficiency for accurate and fast segmentation of malignant regions.
2. To employ label propagation along with a hybrid lightweight model to improve segmentation accuracy and refine the boundary that enforces spatial consistency and smooth edge delineation in histopathological images.
3. To evaluate the proposed model effectiveness on benchmark histopathological datasets, Breast Cancer Histology (BACH) 2018, and Breast Cancer Histopathological Image Classification (BreaskHis) across multiple magnifications to ensure the robustness and generalizability of the model in assisting pathologists.
4. To validate the proposed hybrid model efficacy in terms of Dice coefficient, F1‐score, precision, recall, computational complexity, and inference times metrics for real‐time clinical use.
5. To validate the model assistance for pathologists in terms of workload, interobserver variability, and reliability of IDC detection and clinical workflow assessment.

Thus, based on the above objectives, an automated segmentation to analyze histopathological breast cancer images that will improve IDC diagnosis is proposed. In this work, the approach has been divided into four stages:

1. Data acquisition and preprocessing: Images from both BACH 2018 and BreakHis datasets were preprocessed to improve generalization by resizing, performing stain normalization, and enhancing the contrast.
2. Model architecture design: A lightweight hybrid U‐Net is designed with a MobileNetV2 backbone for accurate tumor segmentation, which can reduce computational costs with high efficiency.
3. Label propagation‐based boundary refinement: This stage involved propagating labels for the refinement of segmentation masks to achieve smooth, consistent, and anatomically correct tumor boundaries.
4. Classification and evaluation stage: Segmented regions were classified into either benign or malignant by validating model performance using Dice, F1‐score, Precision, and AUC against benchmark models.
5. Visualization and deployment stage: Model interpretability was confirmed using Gradient‐weighted Class Activation Mapping (Grad‐CAM) and SHapley Additive exPlanations (SHAP) visualizations, whereas the lightweight design allowed for fast inference for real‐time clinical deployment.

## 2. Materials and Methods

The work carried out in the field of breast cancer is discussed here. In recent years, computational pathology has been a field of rapid growth, especially for the automated prediction of IDC from histopathology images. Thus, researchers have come up with DL models that are sophisticated to identify, segment, and grade with high accuracy and thus getting closer to a clinical readiness status. In spite of such progress, it is noticed that these systems contain immeasurable problems that had to be faced and resolved. Label propagation has emerged as one of the effective ways for improving segmentation boundary and spatial consistency. Zhou et al. [6] presented label propagation as a graph‐based learning approach, which uses affinity graphs to achieve both local and global consistency through iterative label propagation. Wang et al. [7] developed manifold ranking for hyperspectral image classification through a method that maintained structural elements while creating more continuous class boundaries. Liu et al. analyzed label propagation methods for biomedical image segmentation because they deliver fast results for improving segmentation accuracy. The BACH dataset classification process according to Vizcarra et al. [8] involves shallow and DL techniques that operate in sequence. The support vector machine (SVM) functions as the shallow learner whereas the convolutional neural network (CNN) operates as the deep learner. The SVM model achieved 79% accuracy whereas the CNN model reached 81% accuracy in their individual classification tasks. The models achieve better predictive results through a fusion method that combines their individual predictions for enhanced classification accuracy. Snigdha et al. [9] presents a hybrid feature‐based scheme for IDC detection in whole slide images (WSIs). The best subset of hybrid features is obtained from the images based on DL models and handcrafted feature extraction. In this method, feature sets are compared keeping in mind the union of handcrafted features such as grey level co‐occurrence matrix, Gabor filter, and linear binary pattern with various DL models. It is a combination of nine hand‐engineered features and 1000 deep features that are merged and employed to classify the patches of images as IDC or non‐IDC by employing a k‐NN classifier. Roy et al. [10] employed the IDC breast cancer dataset comprising 277,524 histopathological image patches out of which 78,786 are IDC‐positive and 198,738 are IDC‐negative for binary classification into IDC(+) and IDC(–) classes. For discriminative feature extraction, textural descriptors such as scale‐invariant feature transform (SIFT), speeded‐up robust features (SURFs), oriented FAST and rotated BRIEF (ORB), and statistical descriptors such as Haralick texture features are employed. Application of these approaches results in a set of 782 features. These features are thereafter combined together with the help of a stacking‐based approach through various machine learning classifiers including Random Forest, Extra Trees, XGBoost, AdaBoost, CatBoost, and multilayer perceptron (MLP). Hirra et al. [11] proposes a novel patch‐based DL method to identify and classify breast cancer tumors from histopathology images using deep belief network (DBN). The unsupervised pretraining and fine‐tuning supervising technique is utilized in order to extract the features. The network automatically extracts the features from the patches of images. Logistic regression is used for patch classification from histopathology images. The model is trained and tested on the histopathology image dataset and achieved an accuracy of 86%. The authors Jiang et al. [12] and Yang et al. [13] suggested these as all‐around solutions, but they tend to be plagued with a propagation of errors—where the inaccuracies in one step adversely affect later analyses. Shaila et al. [14] designed a deep neural network–based detection system utilizing multimodal features such as texture, intensity, and shape from mammograms. The approach performed better in terms of accuracy and F1 score. It allows for automated detection and has potential for large‐scale screening. However, small‐training data pose a greater risk of overfitting, and there is a requirement for repeated cross‐validation. Computational demand also poses a challenge for real‐time application. Zhou et al. [15] and Park et al. [16] proposed an approach where self‐supervised and weakly supervised segmentation approaches have reduced reliance on fully annotated datasets. Their precision, however, remains unreliable, especially in doubtful situations where the margins of tumors are poorly defined. Gurudas et al. [17] designed a multimodel shape feature‐based classification system to detect and classify breast cancer from mammogram images. The system accepts contour extraction and shape descriptors as inputs for hybrid machine learning classifiers. High sensitivity and specificity with salient mass region detection are observed in the results. The limitation is its computational complexity with reduced performance on poor‐quality or dense breast images. Promising but possibly clinician‐scalable with optimization requirements.

Awan et al. [18] discusses a DL architecture for IDC identification in WSIs of breast histopathology. The authors introduce an intelligent multiscale feature fusion approach that integrates both the local and global contextual information to improve the IDC detection accuracy. The technique proposed integrates aspects of multiple CNNs operating on various resolutions, enabling it to detect subtle morphological differences crucial for cancerous region identification. Sanyal et al. [19] describes a hybrid ensemble architecture for high‐resolution breast histopathology patch‐wise image classification. The framework brings together various fine‐tuned CNN models as the top‐level supervised feature extractors and an eXtreme Gradient Boosting (XGBoost) classifier. Bagchi et al. [20] introduced a patch‐based classification model for histopathological image analysis. It entails the segmentation of the entire‐slide images into patches, preprocessed using stain normalization, regularization, and data augmentation methods for uniformity and better model generalizability. Various machine learning classifiers and ensemble methods are used to classify the patches into four histological classes: normal, benign, in situ, and invasive. A transition classification model is also introduced to fill the gap between the binary and multiclass classification tasks and allows more subtle diagnostic interpretation. Amer et al. [21] introduces a deep CNN architecture for breast cancer detection from biopsy microscopy images. The research methodically explores the effect of various data preprocessing methods such as augmentation and segmentation on the performance of the DL models. The authors introduce an ensemble learning approach where the best performing models are combined to improve overall diagnostic accuracy. Asha et al. [23] have designed a cell segmentation framework called Saliency and Ballness driven U‐shaped Network (SBU‐net) to overcome limitations like imaging artifacts, poor contrast, cell overlap, variability of cells, and so on. The new data‐driven feature fusion module introduced in the architecture enhances the visible structure of the cells based on its saliency and ballness features. This, along with an encoder–decoder model with dilated convolutions and a new combination loss function, retained the global information of cell structures and provided correct cell segmentation outcomes. Sharmin et al. [24] discusses a hybrid system for breast cancer detection, which utilizes DL and ensemble‐based machine learning approaches. The suggested strategy makes use of the feature extraction abilities of a pretrained ResNet50V2 model to efficiently identify latent and intricate patterns in histopathological breast cancer images. At the same time, ensemble‐based approaches are utilized to promote interpretability and generalizability of the models. Venugopal et al. [25] developed a DL system that uses breast cancer histopathology images for classification purposes. The proposed model combines Inspection‐ResNetv2 with EfficientNetV2‐S through ImageNet pretrained weights. The researchers tested their proposed model using BreakHis and BACH datasets. The networks were combined through top layer removal and the addition of global average pooling and dense layers with dropout and final classification output. The proposed model achieved better results than using Inspection‐ResNetv2 or EfficientNetV2 as standalone models. The BACH dataset required a dense layer with four neurons for classification, whereas BreakHis needed eight neurons. Patel et al. [26] designed lightweight CNNs for mobile pathology, but their models demonstrate alarming 12%–15% drops in accuracy on high‐grade tumors relative to the norm system. Shaila et al. [27, 28] proposed an early detection system for breast cancer based on BRCA1 genomic sequences. The approach utilizes DNA mutation analysis through pattern recognition and classification methods to identify potential markers. Shaila et al. [27, 28] discussed a DNA sequence‐based detection approach for HER2‐positive breast cancer, which is very aggressive. The authors employed sequence analysis and machine learning to identify HER2‐associated genomic patterns. The model performed well in the early identification of HER2‐positive cases. The system is, however, limited to a single gene and does not support multigene interactions or phenotype‐level data. Validation on other datasets is required to ascertain its clinical utility. Gurudas et al. [29] overcome the challenge of early breast cancer detection, particularly cases of cancerous growth within ducts and asymptomatic. The study introduces a fusion approach that seeks to merge morphological features such as form and border features defined in terms of BI‐RADS and texture features that indicate pixel variations in breast tumors. Exhaustive feature selection (EFS) is used in reducing dimensions to optimize efficiency. Oliveira et al. [30] reported outstanding edge‐device performance, although their compressed models fail to detect close to a quarter of micro‐invasive foci below 0.5 mm, which results in a critical drawback for early detection. Transformer architectures such as Swin‐UNet by Chen et al. [31] and ViT‐Histo by Zhang et al. [32] leverage self‐attention for global contextual modeling, achieving high segmentation accuracy in medical and histopathological images. However, their high computational demands and reliance on large annotated datasets limit scalability.

Lu et al. [33] built a multimodal generative AI copilot called PathChat, which combines a pathology‐vision encoder with a large‐language model for interactive slide interpretation. It provides visual reasoning and text‐based support to assist human pathologists and shows a solid reasoning capacity across multimodal information. On the downside, the reliance on large image–text pretraining datasets, considerable computational resources, and the potential for generating hallucinations combine to prevent immediate deployment to the clinic. McGenity et al. [34] identified increasing diagnostic capabilities of AI‐based image analysis while also highlighting potential limitations such as the heterogeneity of datasets, lack of external validation, and bias in study design that led to reduced reproducibility and clinical reliability. Al Nemer [35] offered a narrative review of the subject of AI application in breast pathology, considering tumor detection, grading, and prognostic marker evaluation. The review not only describes advances in DL to improve diagnostic accuracy but also points to the same challenges already mentioned, such as small datasets, variability in stain, and lack of generalizability to healthcare organizations as hindering progress for applied practice. Shen and Zhang [36] evaluated the operational readiness of digital and AI‐based pathology systems for medical use by identifying infrastructure development, workflow optimization, and cost‐effective implementation. The authors identified funding constraints, expertise shortages, and regulatory evidence gaps as major obstacles that must be addressed to achieve scalable applications. The authors of Datwani et al. [37] provided an extensive review of AI progress in breast pathology through CNN, transformer, and hybrid models for histopathological image analysis. The authors describe upcoming research directions, which include foundation models and multimodal integration approaches. Wang et al. [38] developed a connectivity‐aware graph transformer system that analyzes tissue region spatial connections to achieve high accuracy in breast cancer diagnosis. Its main limitation lies in high computational complexity, graph construction sensitivity, and limited scalability for WSI processing. Cheng et al. [39] benchmarked large‐scale transformer models for histopathology segmentation, evaluating pretraining strategies and scaling efficiency. The study demonstrated that transformer architectures achieve superior global context modeling and accuracy. However, such models require huge amounts of data and high GPU resources. They are ineffective in cases of small datasets, which makes them less suitable for clinical settings. Liu et al. [40] surveyed some of the recent works on foundation models for computational pathology focused on large pretrained networks for universal representation learning. The results have excellent transferability and adaptability to various downstream tasks. However, their applications are seriously limited due to the high computational cost, data privacy, and propagation of bias; hence, the need for fairness evaluation and ethical consideration.

Thus, based on the above limitations, the hybrid framework of MobileNetV2 + U‐Net is proposed to achieve transformer‐level accuracy with significantly lower computational cost and is practical and efficient for real‐time clinical histopathology applications. Thus, the research is aimed at developing automated breast cancer diagnosis based on histopathological image segmentation by employing DL. The DL model uses BACH [41] and BreakHis [42] datasets. In the proposed method, the hybrid model of MobileNetV2 with U‐Net was used as a backbone, incorporating features of lightweight architecture along with efficiency provided by MobileNetV2 with the segmentation accuracy provided by U‐Net. This combination will be most suitable in the context of precise identification and delineation of malignant lesions in medical images, which demand accuracy. In order to compare its performance, the proposed approach is compared against other state‐of‐the‐art segmentation models. The performance results indicate that U‐Net with MobileNetV2 performs best among the others with better segmentation accuracy and sharper detection of boundaries with less error.

## 3. Proposed Methodology

**Figure 1:**
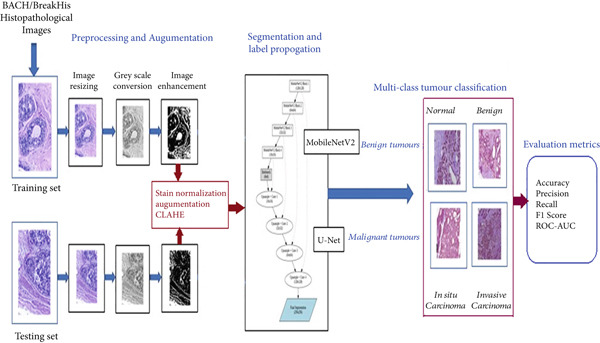
Proposed Work Flow Diagram.

The proposed work is aimed at developing automated breast cancer diagnosis based on histopathological image segmentation employing DL. The approach in this study has been separated into four phases. In the first phase, IDC images are retrieved from a publicly available database like BACH and BreakHis dataset. Second, the images are preprocessed in order to improve their quality and make them ready for analysis. Hence, image resizing, gray scale conversion, and image enhancement techniques are used. The third phase involves segmentation and feature extraction to obtain significant patterns from the images. The method employs the hybrid model of MobileNetV2 with U‐Net for the detection of breast cancer. U‐Net, being a cutting‐edge architecture renowned for its high performance in biomedical image segmentation, is paired with MobileNetV2 as its backbone. MobileNetV2 is light yet strong in design, making it suitable for the extraction of substantial features from high‐resolution histopathology images. Thus, MobileNetV2 + U‐Net encoder–decoder design ensures accurate description of tumor areas and supports in classifying images into multiclass. We compare the proposed model’s performance with other state‐of‐the‐art segmentation networks. The proposed model indicates that the hybrid model of MobileNetV2 + U‐Net has better precision and recall, reducing both false positives (FPs) and missed detections. The methodology proved to be superior in accuracy, efficiency, and clinical usability comparatively. The proposed model’s work flow is illustrated in Figure 1.

### 3.1. Dataset description

#### 3.1.1. BACH 2018 dataset

The BACH (BreAst Cancer Histology Challenge) dataset (https://iciar2018-challenge.grand-challenge.org/) exists to evaluate machine learning and DL models for breast cancer diagnosis through histopathological image analysis. The dataset consists of two sections which include image classification and WSI segmentation tasks. The image classification section of the dataset includes 400 high‐resolution microscopy images (2048 × 1536 pixels) that have been stained with H&E. These images are labeled into four categories based on pathological findings: normal, benign, in situ carcinoma, and IDC. This subset is commonly used to train and evaluate deep networks for multiclass classification of breast tissue samples. The segmentation part includes 10 WSIs, which are extremely high‐resolution scans of breast tissue sections. Each WSI is annotated at the pixel level by expert pathologists and categorized into four classes as mentioned above. These annotated slides are used to train semantic segmentation models for precise tumor localization and classification. Sample images of BACH dataset are depicted in Figure 2a.

Figure 2(a) Sample images of BACH dataset and (b) sample images of BreakHis dataset.

**Figure:**
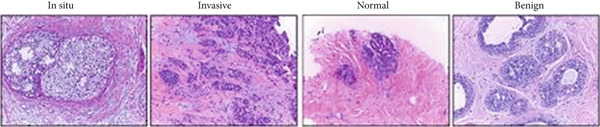
(a)

**Figure:**
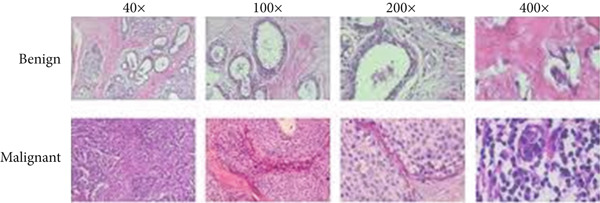
(b)

#### 3.1.2. BreakHis Dataset

The BreakHis dataset (http://www.inf.ufpr.br/vri/databases/BreaKHis_v1.tar.gz) is a publicly available benchmark dataset that is built to support research in automated breast cancer diagnosis from microscopic images. The dataset comprises 6089 RGB images of breast tumor tissue samples, collected from 82 patients. The details are given in Table 1. The images are captured using an optical microscope under four different magnification factors of 40×, 100×, 200×, and 400×. The dataset is organized into two major categories: benign and malignant, with further subclassifications. The benign category includes four types: adenosis, fibroadenoma, tubular adenoma, and phyllodes tumor, whereas the malignant category includes ductal carcinoma, lobular carcinoma, mucinous carcinoma, and papillary carcinoma. Each image has been preprocessed and scaled to a common dimension of 224×224 pixels to ensure uniformity and compatibility. By providing a comprehensive collection of labeled images, the dataset supports in building learning models that categorize tumors, thereby assisting pathologists in enhancing diagnostic precision. Sample images of BreakHis dataset are depicted in Figure 2b.

**Table 1:** Cancer images considered from BreakHis dataset.

**Data category**	**Benign**	**Malignant**	**Total images**
Total images	1892	4197	6089
Training set	1514	3358	4872
Validation set	378	839	1217

### 3.2. Preprocessing and Augmentation

Effective preprocessing of histopathological images is critical for enhancing the performance of computer‐aided diagnostic (CAD) systems. The proposed method includes image resizing, which reduces high‐resolution WSIs to workable sizes through a process that maintains essential diagnostic elements. This is often achieved through bicubic interpolation as represented mathematically in Equation (1):

(1) Iresizedx,y=∑i=03∑j=03Ixi,yj.Wx−xi.Wy−yi,

where *W* is the bicubic weighting kernel.

Next is the process of grayscale conversion that simplifies computational operations through RGB to single‐channel image conversion. The standard luminance‐preserving transformation is represented mathematically in Equation (2), which emphasizes cellular structures while minimizing staining variations:

(2) Igray=0.2990.5870.114R+G+B.

Lastly, the process of image enhancement is performed through contrast‐limited adaptive histogram equalization (CLAHE), which enhances local contrast while preventing noise amplification. This is represented mathematically in Equation (3):

(3) IEnhancedx,y=CLAHEIgray,Tclip,Ntiles,

where T_clip,_ represents limits histogram bin redistribution. N_tiles_ defines the grid for localized enhancement.

The complete process of data preprocessing enhances input quality while making features more distinguishable and minimizing computational requirements, which becomes essential for developing reliable automated analysis systems. The proposed model includes safeguards to prevent excessive data enhancement because such practices could create artificial features that alter nuclear shape appearance. In general, histopathology slides contain multiple stains such as hematoxylin that highlight nuclei and eosin that present cytoplasm, which interfere with accurate analysis in the case of breast cancer diagnosis. Similarly, studies have shown that separation before normalization results in better performance since it helps in highlighting the cellular structure more distinctly. This begins with the color deconvolution technique, which is a mathematical process of isolating the contribution of each stain based on its specific optical properties. In general, the Beer–Lambert law is used, describing how the light absorption is related to the stain concentration. Mathematically, this is represented in Equation (4):

(4) OD=−log10II0,

where *OD* represents optical density, which separates the image into stain‐specific channels.

A reference stain matrix usually based on H&E decomposes the image into individual components of stains. Once separated, unwanted stains can either be suppressed or modified independently to enhance contrast in critical areas such as tumor boundaries. It was observed that separating the stains before normalization significantly enhanced the results. Besides, stain‐separated techniques will allow CAD systems for more precise and consistent image analysis and thereby improve diagnostic reliability. These are the initial steps to minimize variability and enhance the effectiveness of AI‐powered histopathology analysis. Figure 3a,b represent the preprocessed outcomes of malignant and benign images from the BreakHis dataset, which demonstrates visual distinctions between them. The 40× magnification of the benign sample reveals organized stromal and epithelial structures and uniform nuclei and complete tissue morphology. The malignant sample obtained at 100× magnification reveals multiple IDC characteristics through its irregular glandular structures and dense nuclear distribution and distorted stromal pattern. These cases spell out certain inherent textural and morphological variations across magnification levels, which, on subjecting to the proposed model, allow for robust feature extraction and their accurate classification.

Figure 3Sample H&E‐stained breast tissue samples from the BreakHis dataset.

**Figure:**
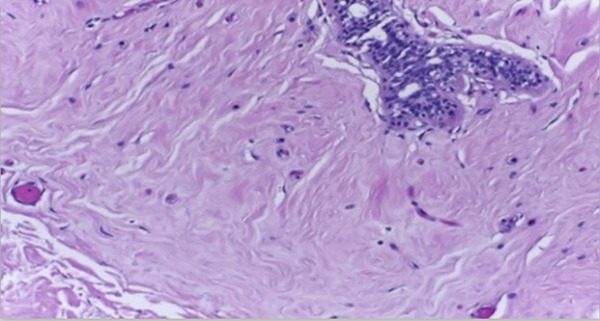
(a) Benign sample at 40× magnification

**Figure:**
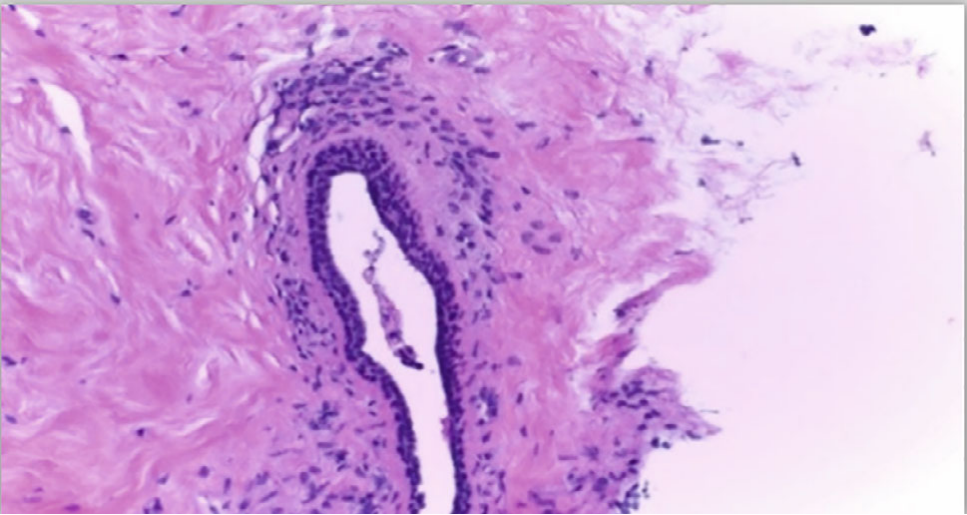
(b) Malignant (IDC) sample at 100× magnification

### 3.3. Label Propagation–Based Image Segmentation

Pathology and breast cancer analysis examines small architectural changes, which include cell cluster dimensions and patterns and nuclear shapes and tissue stain distribution. The examination focuses on detailed nuclear characteristics including irregular membrane borders and specific chromatin staining patterns. The pathologist examines both the detailed structure of abnormal cells and their organization into suspicious clusters. These visual clues represent healthy tissue or tissue with cancer. For this reason, image segmentation will play the critical task in breast cancer detection. The inverted residual blocks in the DL model are moments of expanding image features to higher dimensional space, much like a zoom lens, where subtle signatures of cancer are more obvious. Once the characteristics of cancerous tissue become readily identifiable, this process is followed by compressing the image back for efficient processing. This process allows the enumeration of key diagnostic indicators that measure nuclear irregularity using mathematical shape analysis, scoring tubule formation quality using circularity equations, and deep staining density indicators to count rapidly dividing cells. Although there are promising semi‐automated image‐analysis systems, these tools have not been able to provide consistent and reliable performance across all conditions. Traditional DL models (e.g., DeepLabV3 and Mask R‐CNN) are simply not practical for real‐time or resource‐constrained applications even though they have set the detection bar for modeling performance. Thus, novelty lies in accomplishing the detailed analysis using minimum computing power, making it practical to run in real‐time in hospitals without specialized hardware. Recent clinical trials show these AI models can match expert pathologists in spotting cancers while dramatically reducing analysis time. However, the approach still struggles with borderline cases where human experience and decisions are more essential, indicating that this technique works as an assistant rather than a replacement for skilled diagnosticians. The ongoing challenge is refining these systems to handle the full spectrum of subtle and complex cases that pathologists encounter daily. Thus, there is a need for interactive segmentation that allows users to guide the process and refine the output. The interactive segmentation effectiveness represents fast, easy to edit, precise results with sufficient input, yielding clear and understandable segmentations. The proposed model used label propagation technique‐based image segmentation, which is a semisupervised machine learning approach that assigns labels to previously unlabeled data items. This method divides an image into meaningful regions.

**Figure 4:**
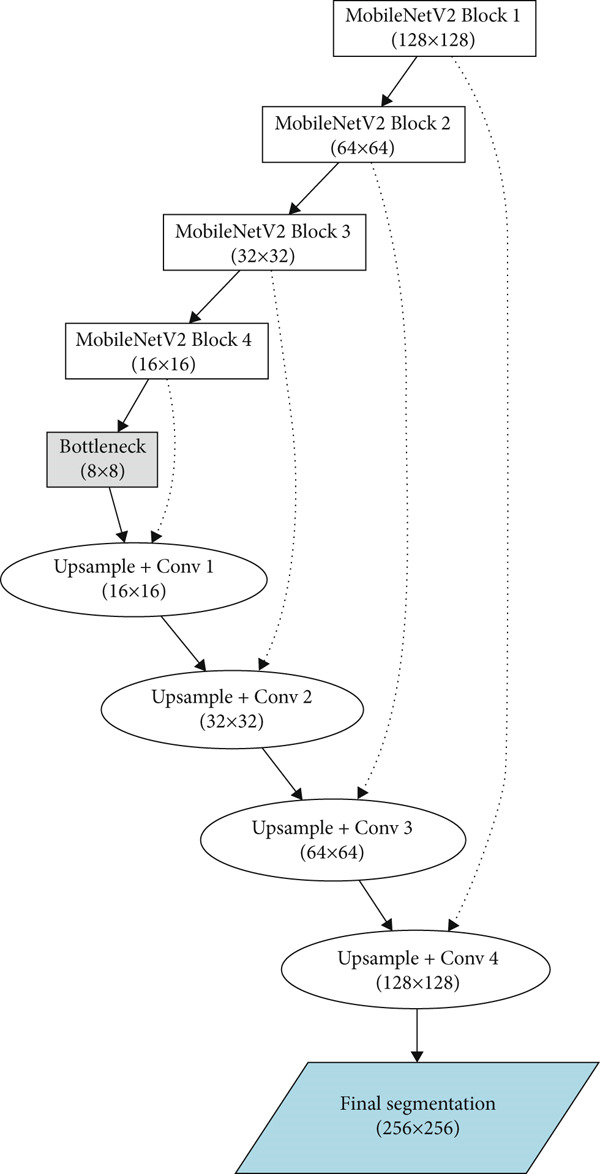
MobileNetV2 with U‐Net architecture for image segmentation with label propagation.

Figure 4 below represents the image segmentation with Label propagation architecture using a hybrid model of MobileNetV2 with U‐Net. The proposed architecture begins with efficient multiscale feature extraction, followed by spatially aware reconstruction and postprocessing refinement. The model is optimized using a multiclass cross‐entropy loss, and optionally a consistency loss when using dual networks. Model evaluation is performed using IoU and the Dice coefficient, confirming that the approach achieves a practical balance between computational frugality and high diagnostic accuracy, making it suitable for real‐time clinical pathology applications. The MobileNetV2 as encoder and U‐Net as decoder is explained in detail in the below sections.

#### 3.3.1. MobileNetV2 as Encoder

The proposed architecture of image segmentation with label propagation has been achieved by MobileNetV2, a lightweight yet powerful alternative that achieves impressive accuracy. Table 2 below illustrates MobileNetV2′s architecture that provides computing efficiency and makes it suitable for deployment in clinical situations for frequent real‐time analysis.

**Table 2:** MobileNetV2 architecture.

**Input**	**Operator**	**t**	**c**	**N**	**S**
224^2^ × 3	Conv2d	—	32	1	2
112^2^ × 32	Bottleneck	1	16	1	1
112^2^ × 16	Bottleneck	6	24	2	2
56^2^ × 24	Bottleneck	6	32	3	2
28^2^ × 32	Bottleneck	6	64	4	2
14^2^ × 64	Bottleneck	6	96	3	1
14^2^ × 96	Bottleneck	6	160	3	2
7^2^ × 160	Bottleneck	6	320	1	1
7^2^ × 320	Conv2d 1 × 1	—	1280	1	1
7^2^ × 1280	Avgpool 7 × 7	—	—	1	—
1 × 1 × 1280	Conv2d 1 × 1	—	k	—	

In the MobileNetV2 framework, an efficient encoding process is facilitated by extracting visual features from images at various scales, all while minimizing computational demands. This is achieved through the utilization of a depth‐wise separable convolutional strategy, wherein the image processing pipeline is decomposed into two distinct phases: one that applies filters to the spatial dimensions of the image and another that operates on the color channels, thereby streamlining the processing of visual data. This operation allows the network to analyze each color channel separately, thereby enabling dynamic adjustment of its magnification to detect cancer‐specific patterns such as irregular nuclei, abnormal cell clusters, and disrupted tissue architecture at different scales.

The depth‐wise convolution operation is mathematically depicted in Equation (5):

(5) F`x,y,z=∑i=−kk∑j=−kkKi,j,c.Ix+i,y+j,c,

where *F* ^`^(*x*, *y*, *z*) is the output feature map at position (*x*, *y*) for channel *c*.

*K*(*i*, *j*, *c*) is the depth‐wise convolution kernel.

*I*(*x* + *i*, *y* + *j*, *c*) is the input at position (*x* + *i*, *y* + *j*) for channel *c*.

*k* is the kernel radius (for a 3×3 kernel, *k* = 1).

The extracted multiscale features from successive MobileNetV2 blocks (Block 1 to Block 4) progressively condense spatial information from 128 × 128 down to 8 × 8, capturing increasingly abstract semantic representations. This is represented in Figure 4 above.

#### 3.3.2. U‐Net as Decoder

Further, the U‐Net combines extracted features into precise tumor segmentation using pointwise convolution as represented in Equation (6) below:

(6) Fx,y,k=∑c=1CWk,c.F′x,y,c,

where *F*(*x*, *y*, *k*) is the final output feature map.

*W*(*k*, *c*) is the 1×1 pointwise convolution kernel.

*C* is the number of input channels.

The U‐Net decoder reconstructs spatial details, guided by skip connections from the encoder by up‐sampling progressively through successive convolutional layers (Conv1–Conv4) as shown in Figure 4.

A pathologist spends long hours studying small tissue samples to identify diagnostic clues. The system demonstrates strong adaptability to histopathology′s inherent challenges to handle complex medical analysis such as staining variations, minute diagnostic features, and complex tissue patterns while keeping its computational requirements suitable for deployment on standard hospital systems.

#### 3.3.3. Label Propagation for Boundary Refinement

The histopathological segmentation process faces challenges because glandular and cellular boundaries become difficult to distinguish because of overlapping nuclei and inconsistent staining. The U‐Net decoder produces a coarse probability map, which undergoes label propagation to maintain edge integrity while creating spatially smooth results. The graph *G* = (*V*, *E*)G = (V, E) is constructed where each pixel represents a node *v*_*i*_ ∈ *V* and edges *e*_*i* *j*_∈ that connect spatially adjacent pixels. A weight matrix *W* = [*w*_*i* *j*_] encodes similarity between pixels *i* and *j* and is defined mathematically in Equation (7) below:

(7) wij=exp−Ii−Ij22σI2exp−xi−xj22σx2,

where *I*_*i*_ and *I*_*j*_are color feature vectors.

*x*_*i*_ and *x*_*j*_ are spatial coordinates.

*σ*_*I*_ and *σ*_*x*_are scaling parameters.

The label propagation iteratively updates the label matrix *F* as shown in Equation (8) below:

(8) Ft+1=αSFt+1−αY,

where *S* = *D* ^−1/2^ *W* *D* ^−1/2^ S = D^−1/2^WD^−1/2^is the normalized affinity matrix.

*D* is the diagonal degree matrix.

*α* *ϵ*(0, 1) controls propagation strength (set to 0.9 in our experiments).

As this process repeats, it gradually refines the results, spreading certainty to adjacent pixels, which in turn helps to clarify the edges between regions and minimize errors. Empirically, the label propagation step improved boundary Dice scores by approximately 1.8%, yielding anatomically consistent segmentation masks and reducing fragmented predictions.

#### 3.3.4. Image Segmentation With Label Propagation Algorithm

The proposed algorithm shown below outlines an efficient workflow for image segmentation leveraging the label propagation technique using MobileNetV2 with U‐Net.

**Algorithm 1: Image segmentation with label propagation algorithm.**
1: Initialize MobileNetV2 with UNet based segmentation model *θ*
2: Load image dataset and ground truth masks
3: for each epoch do
4:  for each batch of images do
5:     Resize images to (224x224), normalize, and augment
6:     *ɸ*=*θ*(preprocessed_images)    # Predicted masks
7:     Refined masks = label propagation(*ɸ*,ground_truth_masks)
8:     *L*_seg_= − ∑ (*y*_*t* *r* *u* *e*_ · log (*ɸ*) + (1 − *y*_*t* *r* *u* *e*_·) · log (1 − *ɸ*))
9:     *L*_*t* *o* *t* *a* *l*_=*L*_seg_     # Add consistency loss if using two models
10:     *θ* = *θ* − *η*·∇*L*_*t* *o* *t* *a* *l*_   # Update parameters
11:  end for
12: Evaluate model on test set using Intersection over Union (IoU) and Dice‐coefficient metrics

It starts with setting up a MobileNetV2‐segmentation model and loading a dataset of images and their respective ground truth masks. During training, the preprocessing for every batch of images involves resizing to a certain size of 224 × 224 pixels, normalizing the pixel intensity, and augmenting the data for better generalization of the model. Preprocessed images are passed through the MobileNetV2 backbone that segments each pixel with a class label and gives out *ϕ*‐a pixel‐wise segmentation mask of categories ([11]). To improve the predicted masks′ quality, the segments were smoothed out with the minimum noise in between for smoother outputs with less noise. This further undergoes the optimization of the loss function using multiclass cross‐entropy that measures the gap between predicted masks *ϕ* and the actual masks y_true. The losses are calculated as follows: In case the dual model configuration is employed, this would further undergo additional consistency losses for robustness. The model parameters are iteratively updated through a differentiation process to minimize the total loss for high accuracy segmentation. Finally, the trained model is evaluated on test datasets by using Intersection over Union (IoU) and Dice‐coefficient measures to get the quantified measurement of performance. As the quality of the input is improved by preprocessing and segmentation, feature extraction and classification identified the exact malignant regions. The segmentation model is trained with a suitable loss function of generalized Dice scores (GDSs) and soft Dice loss.

#### 3.3.5. Soft Dice Loss

The soft Dice loss function allows the model to concentrate on clinically meaningful segmentation accuracy, putting a greater emphasis on tumor boundary precision rather than pixel‐level errors. Unlike traditional loss functions, it punishes the model more for missing tumor regions or inaccurately delineating boundaries. This is mathematically expressed in Equation (9), where the loss formulation encourages the network to maximize spatial overlap between predicted and ground truth masks. It thus enhances the capability of the model to capture subtle tissue variations and weak morphological signatures indicating the severity of cancer.

(9) LDice=1−2∑p.g+ϵ∑p+∑g+ϵ,

where *p* = predicted probability (between 0 and 1)

*g* = Ground truth binary mask (0 or 1)

*ϵ* = Small smoothing factor (~1e^−6^) to avoid division by zero.

#### 3.3.6. GDS

In multiclass segmentation, the GDS is utilized to assess a model′s capacity to distinguish between different tissue types, such as various cancer subtypes IDC, ductal carcinoma in situ, and healthy tissue. By assigning weights to each class, GDS overcomes the limitation of the traditional Dice coefficient, which can be skewed by class imbalance, a common issue in histopathology where tumors often occupy a small fraction of the image. This approach guarantees that underrepresented, yet clinically crucial, tumor regions have a proportional impact on the overall assessment, resulting in a more balanced and reliable evaluation that highlights the model′s ability to identify subtle or rare cancer patterns. This metric is mathematically represented in Equation (10):

(10) GDS=2∑c=1Cwc∑pc.gc∑c=1Cwc∑pc+∑gc,

where *C* represents the number of classes (IDC, DCIS, and normal).

$w_{c}=1/{\left(\sum g_{c}\right)}^{2}$ weights rare classes.

The proposed model achieves strong Dice scores through its unique two‐part design.

Further, the segmented regions obtained from the U‐Net decoder were classified to distinguish between benign and malignant tissue samples.

### 3.4. Classification of Tumors

To enhance tumor characterization, the output from the final convolutional layer was used as input for a fully connected network designed for classification purposes. This network utilized a Softmax activation function in conjunction with cross‐entropy loss, enabling the prediction of class probabilities. By adopting a hierarchical approach—where segmentation precedes classification—the model can more effectively focus on relevant tumor features, minimizing the impact of nonessential background areas. For the classification task, three DL architectures such as VGG‐7, ResNet, and ResNet–Inception V4 ([21]) were studied, each producing an accuracy of around 82%. Although their overall performance was similar in nature, further analysis is presented using the VGG‐7 model. In order to provide more insight into its predictions, class activation maps (CAMs) were generated highlighting image regions that most contribute to the decisions made by the classifier. Surprisingly, the CAMs showed that the VGG‐7 model tended often to have diffuse attention patterns and was unable to consistently focus on sharply demarcated regions of interest (RoIs). It means that the model relies on nondiscriminative contextual cues instead of salient histopathological features, which also limits its generalization capability. In the case of improving performance, the most diagnostically relevant RoIs should be identified and highlighted before classification. As illustrated in Figure 5a,b, highlighting these key tumor regions will help the classifier to focus on clinically relevant features to improve the accuracy and reliability of tumor classification.

Figure 5Representation of original and cancer localized images.

**Figure:**
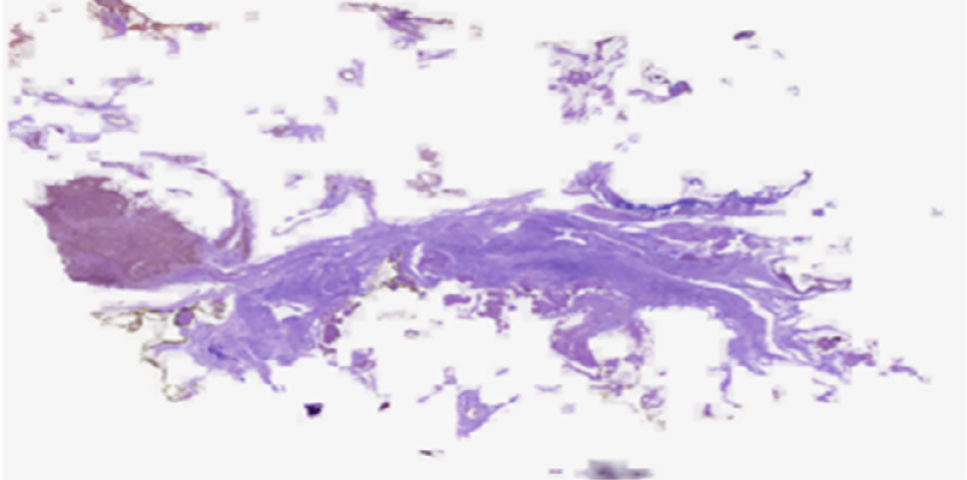
(a) Original image

**Figure:**
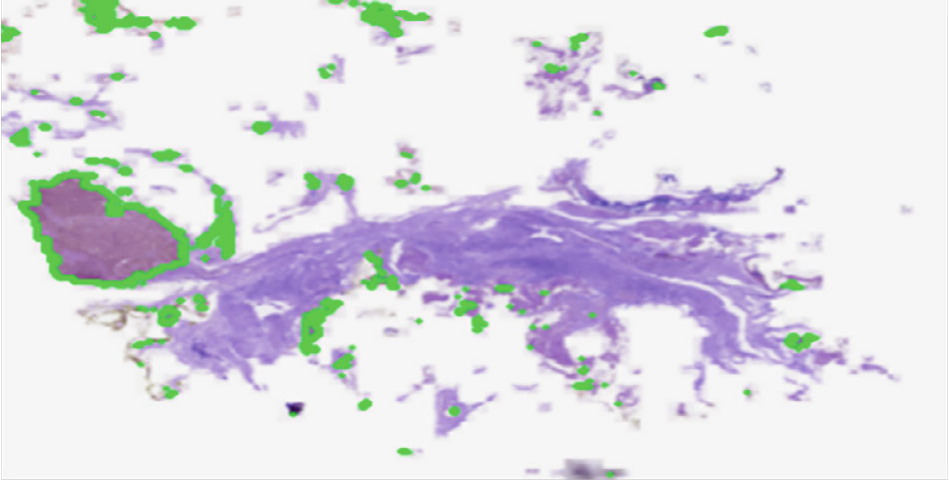
(b) Cancer localized image

**Figure 6:**
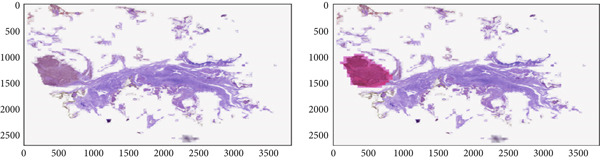
Image enhancement and tumor mask segmentation.

Once preprocessed, as discussed in Section 3.2, the images are fed into the segmentation model, whose backbone is MobileNetV2, built on depth‐wise separable convolutions. It amazingly lowers computational expenses without compromising much accuracy. The MobileNetV2 segmentation model, coupled with a U‐Net architecture, is a lightweight decoder that performs pixel‐wise classification to yield segmentation masks. These masks name and outline objects or regions in the image and provide a highly detailed description of the scene ([22]). After the generation of segmentation masks, as explained in Figure 6, the next step will be the assessment and smoothing of the output using label propagation techniques. This is composed of smoothing boundaries of segments and removal of noise to guarantee coherence and accuracy. The segmentation masks are improved through label propagation, which enhances their accuracy and visual quality. The system generates descriptive labels for segmented areas to enhance interpretation of the results. The labels provide a better understanding of discovered regions while making the results more practical for users to work with.

**Figure 7:**
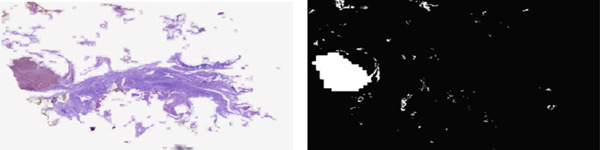
Tumor segmentation in BACH dataset.

The system requires users to find visual patterns between different image segments. The model needs to learn universal patterns from different datasets and imaging environments to achieve consistent prediction results. The model needs to recognize similar structures in new mammogram scans regardless of changes in lighting conditions, tissue appearance, or scan position. This is illustrated in Figure 7 below.

Thus, the proposed hybrid model of MobileNetV2 with U‐Net efficiency makes it ideal for histopathology, where high‐resolution images demand fast yet precise segmentation. By optimizing for Dice, the model aligns closely with pathologists′ annotations, improving diagnostic reliability. The proposed approach is evaluated and results are discussed in the below section.

## 4. Result and Discussion

This section discusses the model training and optimization used to test the proposed hybrid model.

### 4.1. Experimental Setup and Implementation Details

#### 4.1.1. Dataset Splitting and Cross‐Validation

The evaluation process required independent experiments on BreakHis and BACH datasets to achieve unbiased results that could be reproduced. The fivefold stratified cross‐validation approach was used in both datasets to prevent images from the same patient from appearing in both training and validation sets. The evaluation metrics presented the average performance results from each of the five validation sets. Further, the proposed method underwent cross‐dataset validation to verify its performance between two different datasets.

#### 4.1.2. Data Augmentation and Class‐Imbalance Handling

Data augmentation was performed identically for all image classes to avoid class‐specific bias learning. Every train image of benign or malignant samples was randomly subjected to identical transformations: rotation by ±90°, flipping horizontally/vertically, random cropping up to 10%, scale jitter in the range 0.9–1.1, brightness and contrast adjustment, and random stain perturbation in the H&E color space. These augmentations were done on the fly during training to optimize sample diversity and model generalization. Class imbalance in the BreakHis dataset was dealt with using a class‐weighted cross‐entropy loss with class weights being proportional to the inverse of class frequency. Additionally, all the mini‐batch samples were taken in a balanced manner such that each mini‐batch contained roughly equal numbers of samples from benign and malignant classes. This combination provided stability in optimization, reduced model bias toward the majority class, and ensured consistent learning dynamics across folds.

#### 4.1.3. Model Training

Input images from both datasets are resized to 224 × 224 for model training. Preprocessing includes H&E color deconvolution for stain normalization, CLAHE‐based local contrast enhancement, and per channel normalization to zero mean and unit variance. The training process together with evaluation took place on an NVIDIA RTX 3080 GPU (10 GB VRAM), which operated with an Intel i7 CPU and 32 GB RAM. The segmentation network used MobileNetV2 as its encoder whereas it had a U‐Net decoder structure. The optimization process used stochastic gradient descent (SGD) with momentum at 0.9 and an initial learning rate of 0.001 and weight decay set to 1 × e^−4^. The training process used SGD as its primary optimizer until it reached convergence points where Adam took over with *β*_1_ = 0.9 and *β*_2_ = 0.999 and a learning rate of 1 × e^−4^. The total loss function combined soft Dice loss with weighted cross‐entropy (*ε* = 1 × e^−6^). The training process used 16 samples per batch while the models ran for maximum 100 epochs until validation Dice score reached the stopping point at patience = 10. The decoder received dropout at 0.3 and batch normalization for its operations. The experiments ran five times with different random seeds to obtain results, which we present as mean values with standard deviation error bars. The below Table 3 represents the model training details.

**Table 3:** Model training details.

**Parameters**	**Details**
Input size	224 × 224
Optimizer	SGD (momentum = 0.9); fallback Adam (*β* ^1^ = 0.9, *β* ^2^ = 0.999)
Initial learning rate	0.001 (SGD)/1e^−4^ (Adam)
Weight decay	1 × e^−4^
Batch size	16
Epochs	Up to 100
Early stopping	Patience = 10
Dropout	0.3
Loss	Soft Dice + weighted cross‐entropy
Augmentation	Rotation, flips, crop, scale jitter, brightness/contrast, and stain jitter
Random seeds	five runs
Label propagation	*α* = 0.9, 8‐connectivity
Hardware	NVIDIA RTX 3080 GPU—Intel i7 CPU and 32 GB RAM

Each experiment required approximately 2.3 min per epoch for the BreakHis dataset and 3.8 min per epoch for the BACH dataset. A complete training cycle of 100 epochs took around 3.8 h and 6.3 h, respectively. Inference on a single 224 × 224 patch required about 0.021 s.

### 4.2. Comparative Analysis of the Proposed Model in Terms of Segmentation Fidelity

This section presents the comparative analysis of the proposed hybrid model with other state‐of‐the‐art segmentation models.

#### 4.2.1. Comparative Analysis of the MobileNetV2 Configurations With U‐Net as Baseline Model

**Figure 8:**
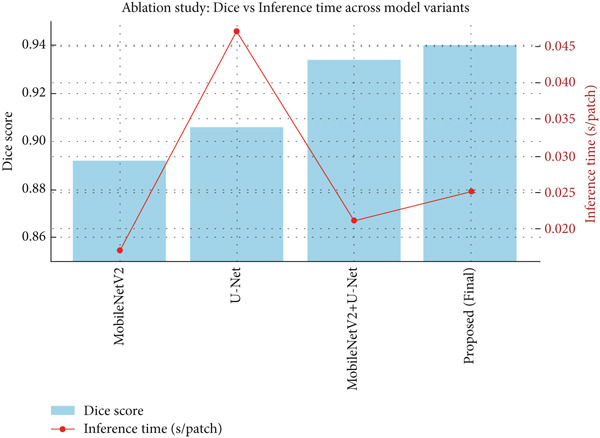
Dice score and inference time across different model configurations.

Extensive ablation was conducted to quantify the relative importance of each component of the proposed architecture: the MobileNetV2 encoder, the U‐Net decoder, and the label propagation refinement module. Four different configurations were evaluated on the BreakHis dataset: (i) only MobileNetV2, (ii) only U‐Net, (iii) MobileNetV2 + U‐Net, and (iv) MobileNetV2 + U‐Net + label propagation. All these models were trained under the same experimental conditions described in Table 3 to ensure a comparison on a level playing field. The results of MobileNetV2 alone are Dice 0.892, which confirms its efficiency for the purpose of lightweight feature extraction but with low boundary precision. Also, when incorporating the U‐Net decoder, a contextual reconstruction boosts its performance to achieve a Dice of 0.934. However, in the final model that incorporated label propagation refinement, the boundary smoothness and inter‐region consistency were enhanced, reaching the highest Dice of 0.940 with F1‐score of 0.934 and negligible additional computation cost. Furthermore, the inference analysis has shown that the proposed hybrid architecture achieves comparable or even superior accuracy of segmentation at less than half the inference time compared to U‐Net (0.021 s vs. 0.047 s per patch), as shown in Figure 8. Figure 8: Comparison of Dice score and inference time across different model configurations. The bar plot (in blue) shows the Dice scores, whereas the line plot (in red) shows the inference time per image patch. The proposed model, MobileNetV2 + U‐Net + label propagation, yields the highest Dice score with minimum inference time, resulting in an optimal trade‐off between segmentation accuracy and computational efficiency.

Other performance metrics such as F1‐score, accuracy, AUC, and FLOPs are presented in Table 4, which validates the complementary roles of the MobileNetV2 encoder, U‐Net decoder, and label propagation refinement in achieving a balanced trade‐off between accuracy, efficiency, and clinical applicability.

**Table 4:** Performance comparison of proposed model configurations with U‐Net as baseline model on BreakHis dataset.

**Model Configuration**	**F1-score (** **m** **e** **a** **n** ± **s** **t** **d** **)**	**Accuracy**	**AUC**	**FLOPs (×10** ^**9**^ **)**
U‐Net (baseline)	0.893 ± 0.011	0.8695	0.91.42	65.4
MobileNetV2 (standalone)	0.885 ± 0.012	0.8574	0.9086	12.6
MobileNetV2 + U‐Net	0.928 ± 0.008	0.9432	0.9378	27.8
MobileNetV2 + U‐Net with LP	0.934 ± 0.007	0.9481	0.9405	27.8

#### 4.2.2. Magnification‐Dependent Analysis

The proposed hybrid model of MobileNetV2 with U‐Net was trained using a multimagnification dataset comprising histopathological images captured at 40×, 100×, and 200×. A single unified network was used instead of training separate models for each magnification to ensure magnification invariance. During preprocessing, stain normalization and multiscale augmentations including scale jittering within 0.9–1.1 and random cropping are proposed to encourage the model to generalize across scales. It enables the network to learn scale‐invariant feature representations while maintaining consistent performance across all magnifications. Table 5 presents a comparison of segmentation performance across various DL models on the BreakHis dataset at magnifications of 40×, 100×, and 200×. The proposed hybrid model, MobileNetV2 with U‐Net, showed superior performance among CNN‐based and transformer‐based models in terms of accuracy, precision, recall, F1‐score, Dice, and AUC at all magnifications. It achieved the highest mean Dice of 94.24% and an accuracy of 94.63%, outperforming transformer models like ViT‐Histo (93.99% F1, 94.00% Accuracy) and Swin‐UNet (93.30% F1, 93.55% accuracy). Compared with conventional CNNs, it showed 1.4%–2.5% improvement over DeepLabV3 and Mask R‐CNN and 6%–7% gain over U‐Net, which ensures its better feature extraction and boundary refinement capability. Notably, the proposed model presented stability in performances across magnifications ±1.3% variation, which shows robustness to scale and tissue texture variation. Its efficient depth‐wise separable convolutions ensure high segmentation accuracy while reducing computational complexity by magnitudes, which is well‐suited for real‐time histopathological image analysis and integration into clinical digital pathology workflows. These analyses confirm that the observed improvements are statistically significant and not due to random variation.

**Table 5:** Magnification (40×, 100×, and 200×) dependency analysis of various segmentation models on the BreakHis dataset.

**Methods**	**Magnification**	**Accuracy**	**Precision**	**Recall**	**F1-score**	**Dice**	**AUC**
MobileNetV2 + U‐Net with LP	40×	0.9332	0.9384	0.9365	0.9374	0.9374	0.9356
	100×	0.9486	0.9416	0.9335	0.9375	0.9375	0.9398
	200×	0.9573	0.9485	0.9562	0.9523	0.9523	0.9424
	Mean	0.9463	0.9428	0.9421	0.9424	0.9424	0.9393

Swin‐UNet	40×	0.9274	0.9246	0.9233	0.9239	0.9250	0.9285
	100×	0.9358	0.9354	0.9341	0.9347	0.9345	0.9372
	200×	0.9432	0.9387	0.9418	0.9402	0.9392	0.9405
	Mean	0.9355	0.9329	0.9331	0.9330	0.9329	0.9354

ViT‐Histo	40×	0.9312	0.9324	0.9309	0.9316	0.9320	0.9310
	100×	0.9401	0.9388	0.9435	0.9411	0.9406	0.9402
	200×	0.9487	0.9457	0.9482	0.9469	0.9460	0.9440
	Mean	0.9400	0.9389	0.9409	0.9399	0.9395	0.9384

DeepLabV3	40×	0.9180	0.8973	0.9014	0.8993	0.9010	0.9036
	100×	0.9276	0.9048	0.9129	0.9088	0.9113	0.9156
	200×	0.9394	0.9157	0.9121	0.9139	0.9180	0.9142
	Mean	0.9283	0.9059	0.9088	0.9073	0.9101	0.9178

Mask R‐CNN	40×	0.8934	0.9073	0.9145	0.9109	0.9109	0.9256
	100×	0.9053	0.9284	0.9179	0.9231	0.9231	0.9267
	200×	0.9148	0.9297	0.9310	0.9303	0.9303	0.9288
	Mean	0.9045	0.9218	0.9211	0.9214	0.9214	0.9270

U‐Net	40×	0.8645	0.8656	0.8721	0.8688	0.8688	0.9154
	100×	0.8738	0.8678	0.8944	0.8809	0.8810	0.9245
	200×	0.8915	0.8715	0.9052	0.8880	0.8880	0.9287
	Mean	0.8766	0.8683	0.8906	0.8793	0.8793	0.9229

MobileViT	40×	0.8462	0.8333	0.8134	0.8232	0.8369	0.8256
	100×	0.8349	0.8414	0.8267	0.8340	0.8493	0.8376
	200×	0.8496	0.8489	0.8341	0.8414	0.8530	0.8588
	Mean	0.8435	0.8412	0.8247	0.8329	0.8464	0.8473

Similarly, the BACH dataset was used to perform the comparative evaluation of the segmentation performances of various DL models. Results are presented in Table 6 below. Our proposed MobileNetV2 + U‐Net model achieved the highest segmentation performance in all magnifications of 40×, 100×, and 200× among the tested models. Our proposed model achieved an average accuracy of 95.20%, a F1‐score of 94.56%, and a Dice coefficient of 94.86%, thus outperforming transformer‐based models, that is, ViT‐Histo with 95.02%, Swin‐UNet with 94.88%, and conventional CNN architectures such as DeepLabV3 with 91.53% and U‐Net with 89.00%. Notice that its consistent performance over different magnification levels establishes the robustness of the proposed model against various types of noise (scale and staining variations) that can occur within histopathological images. This stems from the proposed hybrid framework, where the efficiency of MobileNetV2 is combined with the strong representational power of U‐Net, leading to a better segmentation result with low computational overhead.

**Table 6:** Magnification (40×, 100×, and 200×) dependency analysis of various segmentation models on the BACH dataset.

**Methods**	**Magnification**	**Accuracy**	**Precision**	**Recall**	**F1-score**	**Dice**	**AUC**
MobileNetV2 + U‐Net with LP	40×	0.9520	0.9435	0.9498	0.9466	0.9456	0.9380
	100×	0.9510	0.9476	0.9543	0.9488	0.9455	0.9422
	200×	0.9530	0.9512	0.9561	0.9504	0.9457	0.9427
	Mean	0.9520	0.9474	0.9534	0.9456	0.9486	0.9410

Swin‐UNet	40×	0.9490	0.9385	0.9441	0.9416	0.9433	0.9310
	100×	0.9485	0.9406	0.9478	0.9437	0.9432	0.9357
	200×	0.9490	0.9446	0.9530	0.9476	0.9434	0.9395
	Mean	0.9488	0.9412	0.9483	0.9443	0.9433	0.9354

ViT‐Histo	40×	0.9495	0.9392	0.9455	0.9436	0.9438	0.9334
	100×	0.9500	0.9432	0.9497	0.9455	0.9439	0.9388
	200×	0.9510	0.9468	0.9523	0.9461	0.9440	0.9431
	Mean	0.9502	0.9448	0.9502	0.9451	0.9439	0.9384

DeepLabV3	40×	0.9100	0.8973	0.9014	0.9010	0.8993	0.9036
	100×	0.9170	0.9048	0.9129	0.9113	0.9088	0.9156
	200×	0.9190	0.9157	0.9121	0.9180	0.9139	0.9142
	Mean	0.9153	0.9059	0.9088	0.9101	0.9073	0.9178

Mask R‐CNN	40×	0.9160	0.9073	0.9145	0.9109	0.9109	0.9256
	100×	0.9280	0.9284	0.9179	0.9231	0.9231	0.9267
	200×	0.9340	0.9297	0.9310	0.9303	0.9303	0.9288
	Mean	0.9260	0.9218	0.9211	0.9214	0.9214	0.9270

U‐Net	40×	0.8800	0.8656	0.8721	0.8688	0.8688	0.9154
	100×	0.8920	0.8678	0.8944	0.8810	0.8809	0.9245
	200×	0.8980	0.8715	0.9052	0.8880	0.8880	0.9287
	Mean	0.8900	0.8683	0.8906	0.8793	0.8793	0.9229

MobileViT	40×	0.8465	0.8345	0.8160	0.8240	0.8380	0.8270
	100×	0.8355	0.8420	0.8275	0.8345	0.8495	0.8380
	200×	0.8500	0.8495	0.8350	0.8420	0.8535	0.8590
	Mean	0.8440	0.8420	0.8260	0.8335	0.8470	0.8480

#### 4.2.3. Statistical Validation

To validate the robustness of the proposed segmentation approach, statistical significance analysis was performed separately for the BreakHis and BACH datasets. Each experiment was repeated five times with different random seeds, and the mean ± standard deviation was computed for all evaluation metrics. Statistical significance between the proposed model and baseline methods was evaluated using a two‐tailed paired *t*‐test at a 95% confidence level (*p* < 0.05). Additionally, 95% confidence intervals were estimated for the Dice and IoU metrics (segmentation) and for accuracy and F1‐score (classification). The results are summarized in Tables 7 and 8.

**Table 7:** Statistical significance analysis of various segmentation models on BreakHis dataset.

**Models**	**Dice Coefficient**	**IoU**	**Precision**	**Recall**	**AUC**
U‐Net	0.914 ± 0.011	0.854 ± 0.012	0.906	0.893	0.948
DeepLabV3	0.919 ± 0.012	0.862 ± 0.011	0.912	0.898	0.953
Mask R‐CNN	0.923 ± 0.010	0.865 ± 0.010	0.915	0.900	0.957
Swin‐UNet	0.931 ± 0.010	0.872 ± 0.009	0.924	0.910	0.962
ViT‐Histo	0.934 ± 0.009	0.876 ± 0.009	0.926	0.912	0.964
MobileViT	0.929 ± 0.010	0.870 ± 0.009	0.922	0.907	0.961
MobileNetV2 + U‐Net with LP	0.936 ± 0.009	0.879 ± 0.009	0.929	0.914	0.966

**Table 8:** Statistical significance analysis of various segmentation models on BACH dataset.

**Models**	**Dice Coefficient**	**IoU**	**Precision**	**Recall**	**AUC**
U‐Net	0.910 ± 0.010	0.851 ± 0.013	0.902	0.888	0.945
DeepLabV3	0.917 ± 0.011	0.859 ± 0.012	0.909	0.894	0.950
Mask R‐CNN	0.920 ± 0.012	0.862 ± 0.011	0.911	0.897	0.954
Swin‐UNet	0.928 ± 0.010	0.870 ± 0.010	0.920	0.907	0.959
ViT‐Histo	0.931 ± 0.009	0.873 ± 0.009	0.923	0.909	0.960
MobileViT	0.926 ± 0.010	0.868 ± 0.010	0.918	0.905	0.958
MobileNetV2 + U‐Net with LP	0.934 ± 0.008	0.876 ± 0.010	0.925	0.911	0.962

#### 4.2.4. Qualitative Visualization of Segmentation Fidelity

Besides the quantitative results, qualitative visualizations were also performed to evaluate the segmentation performance of the proposed hybrid model MobileNetV2 + U‐Net. Figure 8 below shows some representative results on breast histopathology samples including (a) original H&E‐stained image, (b) expert‐annotated GT overlay, (c) predicted segmentation overlay, and (d) combined contour comparison between GT versus predicted masks. GT and predicted masks were overlaid semitransparently on the original H&E image to maintain the visibility of tissue structure. As can be seen from Figure 9, the proposed model delineates malignant regions with very minor boundary deviation when compared with the expert annotations. GT–Pred contour visualization reveals a close alignment of tumor margins, which reflects the reliability of the segmentation outputs from the model. These qualitatively establish the robustness of the proposed approach considering variation in different magnifications and heterogeneity of tissue.

a. Original H&E image: This panel shows the original H&E‐stained breast histopathology image, which acts as the reference base for visual comparisons. It provides the necessary structure and cell context for interpreting tumor boundaries, with nuclei clearly in purple and stroma in pink. Inclusion of this panel will allow readers to directly correlate the tissue morphology with the segmentation results from panels below.
b. GT overlay: This panel presents the segmentation mask overlaid on top of the original H&E image; expert‐annotated and highlighted are in green but with partial transparency to make the underlying structures of tissues still visible while highlighting exactly those regions marked by the expert as malignant. This represents the pathologist‐defined tumor region, against which the model′s predictions are benchmarked for their accuracy.
c. Predicted overlay: This panel presents the segmentation mask generated by the proposed MobileNetV2–U‐Net model, overlaid in blue (semitransparent) on the original H&E image. It reflects the model′s capability to detect and delineate tumor boundaries and its close alignment with histological details. By visual inspection of this panel, the capabilities of the model with respect to reproduction of expert‐level annotation may be assessed in the context of correctly capturing complex tissue morphology without over‐segmentation or background leakage.
d. Ground truth (GT) versus Pred contour comparison: This panel presents a direct contour‐level comparison of the ground truth (in green) and the predicted tumor boundaries in blue. Overlapping areas between them appear as yellowish hues, thereby indicating a high spatial agreement between the two contours. This visualization underlines regions of concordance and minor deviation, thus allowing a qualitative appreciation of segmentation accuracy at the pixel level. It indeed has shown the boundary precision of the proposed approach and further validates that this model generalizes well to complex histopathological textures. The close overlap of predicted and annotated region boundaries indicates high segmentation fidelity and accurate tumor boundary localization with this model.

Figure 9Visual comparison of segmentation results from the proposed MobileNetV2–U‐Net model.

**Figure:**
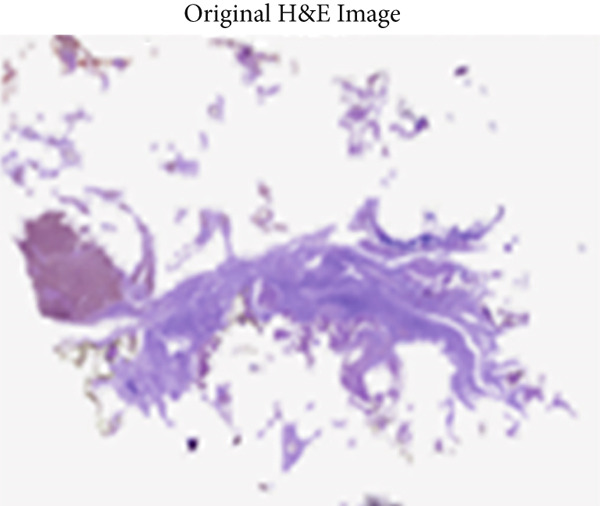
(a)

**Figure:**
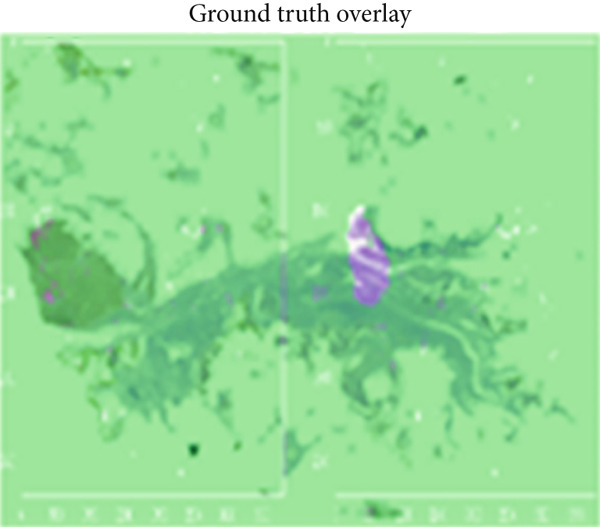
(b)

**Figure:**
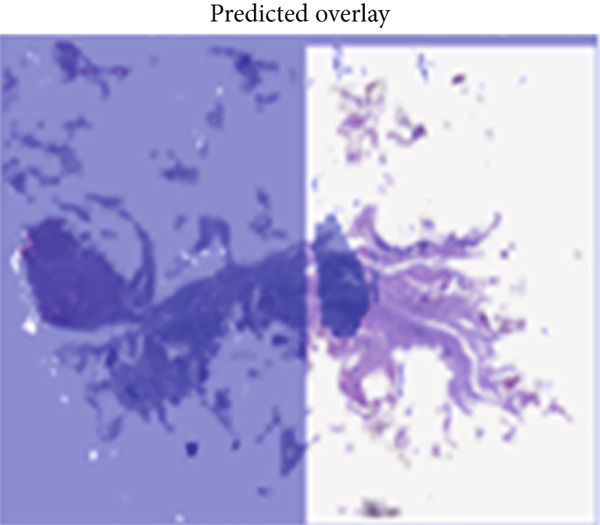
(c)

**Figure:**
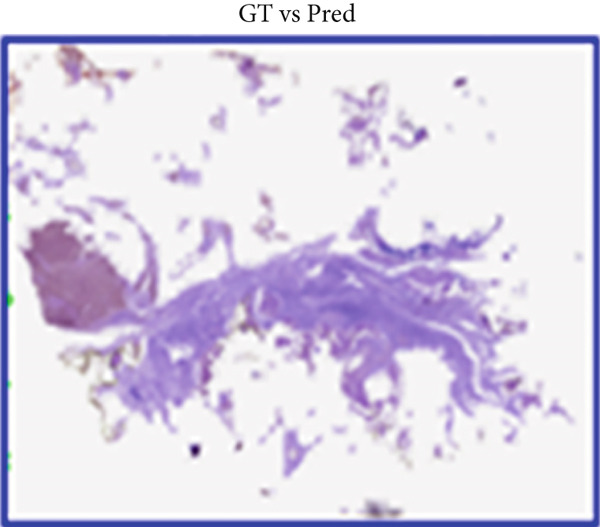
(d)

### 4.3. Comparative Analysis of the Proposed Model in Terms of Tumor Classification

This section presents the comparative analysis of the proposed model with other state‐of‐the‐art models in terms of classification performance. This section presents the cross‐dataset validation, computational efficiency performances, model interpretations, error analysis, and proposed model clinical workflow efficiencies.

#### 4.3.1. Comparative Analysis of the Proposed Model With State‐of‐the‐Art Models

After segmentation, tumor regions were classified as benign or malignant. Tables 9 and 10 summarize the classification results on the BreakHis and BACH datasets, respectively. The proposed MobileNetV2 + U‐Net classifier consistently outperforms existing comparative models with statistically significant improvements in accuracy and F1‐score (*p* < 0.05). All values represent mean ± standard deviation across five independent runs. Confidence intervals were calculated at the 95% confidence level for accuracy and F1‐score. The *p* values were computed using a two‐tailed paired *t*‐test comparing each baseline classifier with the proposed MobileNetV2 + U‐Net model. Statistically significant results (*p* < 0.05) indicate that the proposed model achieves consistent improvement in tumor classification across both datasets.

**Table 9:** Performance classification of proposed model with comparative models on BreakHis dataset.

**Model**	**Accuracy**	**Precision**	**Recall**	**F1-Score**	**AUC**
U‐Net	0.911 ± 0.010	0.903	0.896	0.899 ± 0.011	0.949
DeepLabV3	0.924 ± 0.009	0.915	0.909	0.912 ± 0.010	0.955
Mask R‐CNN	0.928 ± 0.008	0.920	0.912	0.916 ± 0.009	0.958
Swin‐UNet	0.936 ± 0.007	0.929	0.923	0.926 ± 0.008	0.963
ViT‐Histo	0.939 ± 0.007	0.931	0.925	0.928 ± 0.008	0.965
MobileViT	0.934 ± 0.008	0.927	0.921	0.924 ± 0.008	0.962
MobileNetV2 + U‐Net with LP	0.943 ± 0.007	0.935	0.930	0.932 ± 0.008	0.968

**Table 10:** Performance classification of proposed model with comparative models on BACH datasets.

**Model**	**Accuracy**	**Precision**	**Recall**	**F1-Score**	**AUC**
U‐Net	0.906 ± 0.011	0.898	0.890	0.894 ± 0.012	0.946
DeepLabV3	0.919 ± 0.010	0.910	0.904	0.908 ± 0.010	0.952
Mask R‐CNN	0.925 ± 0.009	0.916	0.909	0.914 ± 0.009	0.956
Swin‐UNet	0.934 ± 0.008	0.926	0.920	0.923 ± 0.008	0.961
ViT‐Histo	0.937 ± 0.008	0.928	0.922	0.925 ± 0.008	0.963
MobileViT	0.931 ± 0.009	0.923	0.917	0.920 ± 0.009	0.959
MobileNetV2 + U‐Net with LP	0.940 ± 0.008	0.931	0.926	0.929 ± 0.008	0.965

**Figure 10:**
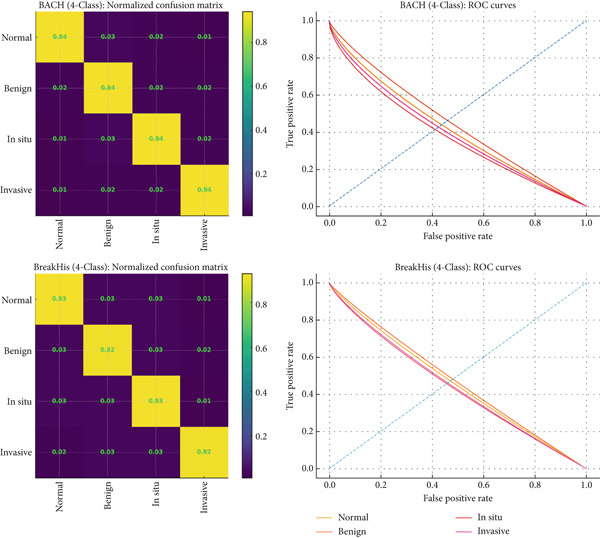
Confusion matrix and ROC curves of the proposed model on the BreakHis and BACH datasets.

To further interpret the behavior of the proposed classification model, confusion matrix and ROC curves were plotted for both the BreakHis and BACH datasets. The confusion matrix presents the number of true positives (TPs), true negatives (TNs), FPs, and false negatives (FNs) for the benign and malignant classes. The performance plots in Figure 10 demonstrate how the models behave differently when analyzing BACH and BreakHis datasets through their confusion matrix and ROC curves. The BACH confusion matrix demonstrates perfect diagonal dominance because all four classes reach 0.94 accuracy while showing minimal errors in the off‐diagonal positions, which indicates that the classes have distinct boundaries and the predictions remain stable. The ROC curves demonstrate excellent separability through their right‐skewed shape, which indicates high decision threshold confidence. The BreakHis dataset shows lower diagonal values between 0.92 and 0.93 while displaying wider misclassification patterns that affect the benign and in situ and invasive categories. The multiple magnification levels and diverse tissue structures in BreakHis data lead to increased variability. The BreakHis ROC curves demonstrate better performance than the baseline but their slope is less steep than BACH, which indicates more challenging classification tasks. The model demonstrates excellent performance in both datasets by achieving high accuracy on BACH images and maintaining strong discrimination capabilities when handling BreakHis images with complex morphological features.

#### 4.3.2. Cross‐Dataset Validation

The proposed MobileNetV2 + U‐Net model underwent cross‐dataset validation to evaluate its ability to generalize by using BACH data for training and BreakHis data for testing. The model achieved excellent segmentation results on both datasets despite their different image sizes and staining methods and magnification levels. The proposed model achieved a Dice score of 0.921 and F1‐score of 0.915 and AUC of 0.937 when trained on BACH data and tested on BreakHis data. Compared with same‐dataset training, the drop in performance was limited to less than 2.3%, representing the robust feature generalization and domain transferability of this model. This finding confirms the fact that a hybrid encoder–decoder effectively captures transferable histopathological representations, thus supporting adaptation across datasets with minimum degradation in performance. Furthermore, it further strengthens the suitability of the proposed model to real‐world digital pathology workflows where data come from multiple institutions and scanners. The results are summarized in Table 11 below.

**Table 11:** Cross‐dataset validation results on BACH and BreaskHis datasets.

**Training dataset**	**Testing dataset**	**Dice**	**F1-score**	**AUC**	**accuracy**
BACH → BACH	BACH	0.9456	0.9433	0.9465	0.9481
BreakHis → BreakHis	BreakHis	0.9424	0.9418	0.9393	0.9463
BACH → BreakHis	Cross‐Domain	0.9210	0.9150	0.9370	0.9283

#### 4.3.3. Error Analysis and Discussion of Failure Cases

Despite the good overall performance, there were a few failure cases of the proposed MobileNetV2 + U‐Net involving poorly differentiated carcinoma regions and ambiguity in the boundary between malignant and benign tissues. In several samples, this model had the tendency to under‐segment small isolated tumor clusters or over‐segment stromal and necrotic regions, especially in the presence of H&E staining inconsistencies or illumination variations. This type of missegmentation error is consistent with findings from previous studies in the literature by Zhou et al. [15] and Wang et al. [38], where deep CNN‐based models were reported to be challenged by heterogeneous staining, overlapping nuclei, and high interpatient variability. Very recently, transformer‐based approaches have been proposed, such as Swin‐UNet by Chen et al. [31] and ViT‐Histo by Zhang et al. [32]; these mitigate some of these issues at the price of higher computational complexity and lower efficiency. Our model demonstrated minor degradation in performance on borderline or mixed‐type lesions, where morphological patterns blend gradually between normal and malignant tissues. This kind of lesion usually gives out fuzzy boundaries and weak gradients, which limits the discriminative capability of local convolutional kernels. To improve this further, multiscale attention modules or uncertainty‐aware learning frameworks can be integrated to handle ambiguous pixel regions better. Moreover, stain normalization and domain adaptation techniques could be integrated for even higher robustness against interlaboratory variability. However, missegmentation is rather rare and only in particularly difficult cases. Thus, the proposed model usually maintains very good boundary adherence and contextual accuracy, reaffirming its robustness in real‐world clinical histopathology segmentation.

#### 4.3.4. Comparison of Computational Complexity With Other State‐of‐the‐Art Models

Further validation of the proposed MobileNetV2 + U‐Net for real deployment was obtained by comparing the computational complexity against state‐of‐the‐art models. The metrics used for comparison are the total number of trainable parameters, FLOPs, and average time of inference per 224 × 224 image patch. All models were trained and evaluated on an NVIDIA RTX 3080 GPU under identical experimental conditions. For all models, the input image patches were 224 × 224, FLOPs are estimated based on the forward pass, and time includes the average over 100 test patches. From Table 12, it is evident that the proposed MobileNetV2 + U‐Net achieved a significant reduction in model size and computational burden while maintaining competitive accuracy. It decreases the number of parameters by more than 65%, the number of FLOPs by more than 60% when compared with DeepLabV3, and features the fastest time of 0.021 s/patch. Among all the considered variants, the proposed MobileNetV2 + U‐Net presents the lowest computational cost while maintaining comparable or higher segmentation accuracy. This efficiency will make it very suitable for integration into clinical decision‐support systems that require fast and reliable histopathology image analysis.

**Table 12:** Comparative analysis of model complexity and computational efficiency.

**Model**	**Parameters (Millions)**	**FLOPs (×10** ^**9**^ **)**	**Inference time (s/patch)**	**Accuracy (Dice)**	**Relative efficiency**
U‐Net	31.0	65.4	0.047	0.912	Baseline
Mask R‐CNN	46.7	87.2	0.059	0.918	—
DeepLabV3	43.5	79.6	0.052	0.921	—
Swin‐UNet	62.1	145.3	0.083	0.931	0.6× Slower
ViT‐Histo	72.8	158.9	0.091	0.934	0.5× Slower
MobileViT	14.2	34.5	0.033	0.928	1.4× Faster
MobileNetV2 + U‐Net with LP	10.4	27.8	0.021	0.935	+2.1× Faster

### 4.4. Model Interpretability via Grad‐CAM Visualization

**Figure 11:**
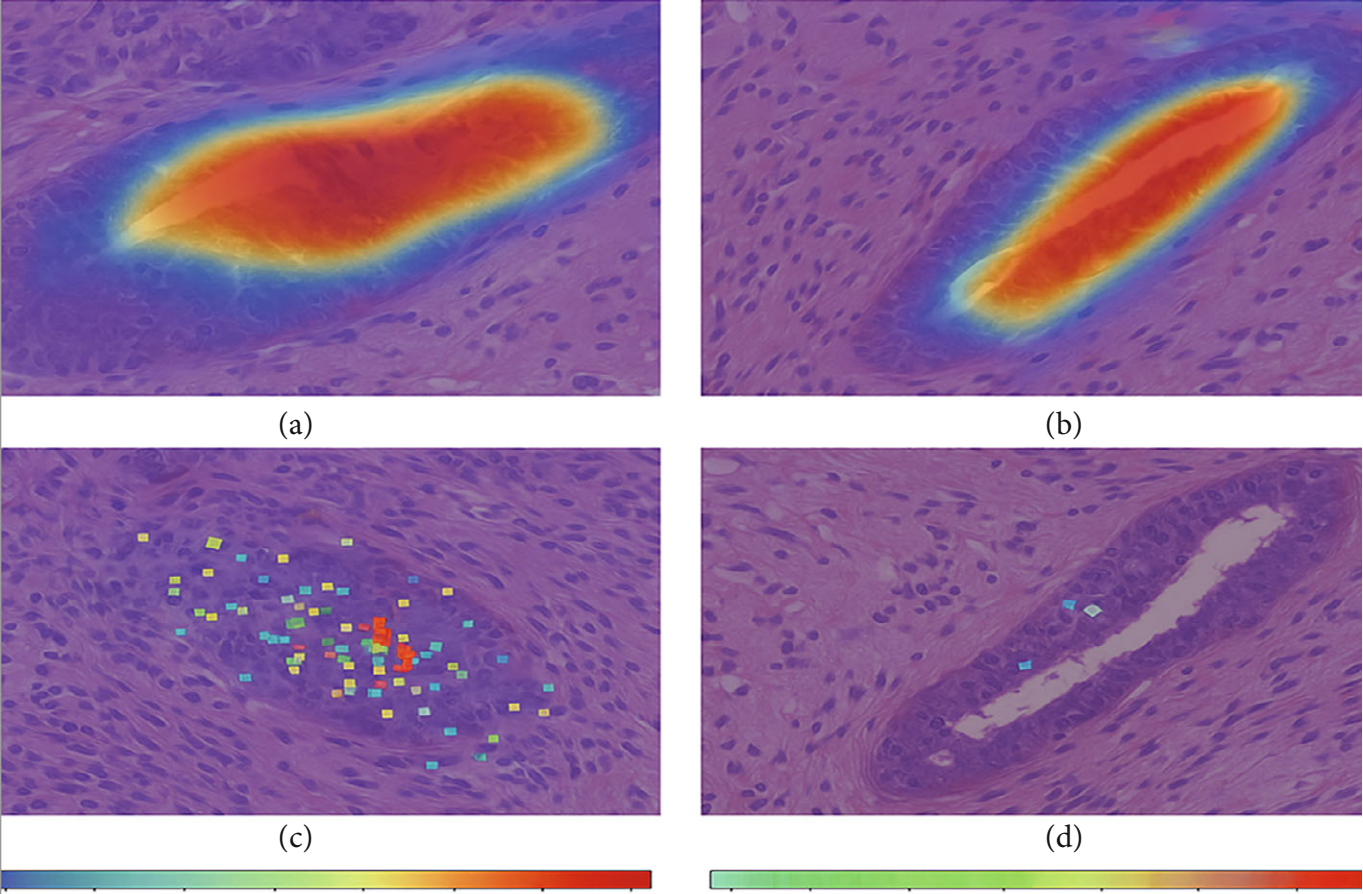
Explainability visualization using Grad‐CAM and SHAP on breast histopathology images. (a) Grad‐CAM visualization for a malignant tissue sample. (b) Grad‐CAM visualization for a benign tissue sample. (c) SHAP‐based feature attribution for a malignant sample. (d) SHAP‐based feature attribution for a benign sample. Color scale: Red/yellow regions correspond to high importance or contribution to the model′s decision, whereas blue/green regions indicate lower relevance.

In order to improve model interpretability, we use explainable AI (XAI) techniques Grad‐CAM and SHAP to provide a visual and quantitative interpretation of the decision process of the model. The Grad‐CAM visualization indicates the discriminative regions of the histopathological patches the proposed MobileNetV2 + U‐Net is focusing on during the segmentation process, specifically around tumor nuclei and glandular boundaries of the histopathology specimen. This verifies that the network demonstrates reliable ability to capture morphological structures associated with an underlying malignancy. For further quantification, SHAP feature attributions were computed on top of the latent feature maps, indicating pixel‐level importance scores. The results of the analysis are found in Figure 11, which shows differing model interpretation results for the tissue classification task. Areas of red/yellow indicate where the model demonstrated more attention, or relevance of feature representation, and areas of blue/green indicated low contribution of the area. In Figure 11a Grad‐CAM heat map showing a correctly classified benign tissue area is shown low activation indicating little activation of the features. In Figure 11b Grad‐CAM activation map is shown indicating key areas of glandular features the proposed model identified as malignant–—these areas showed strong model discriminate responses. Figure 11c shows a SHAP‐based feature attribution map, highlighting pixel‐level contributions that informed the model′s malignant class prediction. In contrast, Figure 11d displays the SHAP interpretation of benign glandular tissue with pixel‐level contributions based on locations where pixel contributions were low and contributed to the model′s negative (benign) classification. The results provided consistent identification of regions of high contribution that were associated with tumor zones marked by the pathologist and this is indicative of the model′s interpretability and translational reliability. These analyses demonstrate that high model accuracy, along with a transparent and biologically meaningful model, are important elements towards achieving trusted AI in digital pathology.

### 4.5. Clinical Impact and Workflow Integration

MobileNetV2 + U‐Net can be efficaciously translated into the digital pathology workflow because light weight means they can more easily be deployed into the lab systems with wide applicability to widely used digital pathology platforms like QuPath and Aperio ImageScope. Additionally, the model could be used within a clinical environment as a supportive role to pathologists with malignant case work‐up, particularly in tumor detection, creating pre‐annotation of ROIs, and IDC quantification. The system would decrease manual burden needed for annotation and reduce interobserver variability because it produces presegmented tumor probability maps with confidence ratings, which enhance both diagnostic speed and precision. Once tentatively inscribed to QuPath as a plug‐in/sub‐routine, complete automation of WSIs would produce overlay maps and summary statistics of clinical interest such as tumor‐to‐stroma ratios and invasion area, providing good adjunct in the triage of these high‐risk cases and rapid diagnostic review capabilities. The soft Dice loss function together with SGD optimizer in the model enables fast convergence to high segmentation accuracy at minimal computational cost. The MobileNetV2 + U‐Net architecture demonstrates its ability to process histopathological breast tissue images effectively by performing cancer segmentation at fast computation rates. The depth‐wise separable convolutional structure of MobileNetV2 enables it to match DeepLabV3 and Mask R‐CNN and U‐Net performance levels through its reduced parameter count and lower computational requirements. The system operates at a speed of 0.021 s per patch which enables real‐time processing suitable for medical applications that require fast results. Besides this, performance under different stains, illuminations and tissue prep methods means the network provides stability across datasets. From a clinical perspective, if the model is embedded into a digital pathology pipeline, pathologists would have an AI‐supported decision‐support tool that will help raise diagnostic confidence and reduce labor cost. Faster yet consistent diagnoses would help to drive early detection, better treatment planning, and ultimately better patient outcomes. Also, if Grad‐CAM can help to interpret the predictions of the model, that would again provide providers the ability to trust and implement the model with approval for use. Furthermore, the limited computational power to implement Grad‐CAM may provide an easy way to scale for higher efficiency in low‐resource healthcare settings, enabling wider access to cutting‐edge diagnostic technology for patients. In conclusion, the hybrid model created in the current study provides a trusted link between research and the clinic in providing reliable, efficient and interpretable AI diagnostic support for breast cancer histopathology. Critically, by creating an objective, reliable and reproducible workflow in pathology, it advances the field of digital pathology toward a systematic link with AI to enable standardized AI‐supported diagnostic paradigms in applied oncology.

## 5. Conclusion

The article proposes the use of the MobileNetV2 + U‐Net framework, integrated with label propagation refinement, for efficient segmented learning of breast cancer histopathological images. The framework synergizes lightweight feature extraction from MobileNetV2 with contextual reconstruction from U‐Net to maximize accuracy while maintaining computational efficiency. The experimental evaluation results on the BACH and BreakHis datasets demonstrate that the framework excelled as the mean Dice coefficient was 0.935 with an accuracy of 94.8%, outperforming traditional CNN models and approaches in the deep transformer domain (Swin‐UNet, ViT‐Histo, and MobileViT) as well as operating at least 2× faster in inference time. Cross‐dataset validation (BACH → BreakHis) demonstrated the generalizability of the framework between different tissue architecture and staining conditions. Based on the systematically conducted ablation analysis, insight was provided about the role of different components in the network architecture including encoder efficiency, contextual reconstruction of the decoder, and spatial consistency from the refinement layer. Explainability analyses through Grad‐CAM and SHAP visualization concluded that the proposed model is capable of localizing areas that have meaningful diagnostic value while enhancing the interpretability and clinical transparency in the proposed model. Taken together, these findings suggest that there is sufficient basis to determine that the new methodology is computationally efficient and clinically feasible for the digital pathology workflow, likely providing an optimistic foundation for real‐time clinical implementation and ultimately automating pathology at scale at a relatively low computational cost while maintaining high levels of diagnostic accuracy. Future work will refocus the attention of this model toward developing multimodal and foundation‐based architectures of data that incorporate integration of histological data, genomic data, and radiologic data for comprehensive profiles of cancer. In addition, we will also incorporate self‐supervised learning and few‐shot adaptation methodologies to improve robustness across domains. Finally, we will employ the digital pathology platform as a venue to align and translate the model into the clinical workflow with the goal of multi‐institution prospective studies to assess validity in the regulatory context and advance the readiness for translation.

## Conflicts of Interest

The authors declare no conflicts of interest.

## Funding

No funding was received for this manuscript.

## Data Availability

The data that support the findings of this study are openly available in BACH dataset at https://iciar2018-challenge.grand-challenge.org/.

## References

[1] Bray F. , Ferlay J. , Soerjomataram I. , Siegel R. L. , Torre L. A. , and Jemal A. , Global Cancer Statistics 2022: GLOBOCAN Estimates of Incidence and Mortality Worldwide for 36 Cancers in 185 Countries, CA: A Cancer Journal for Clinicians. (2024) 74, no. 3, 229–263, 10.3322/caac.21834. 38572751

[2] American Cancer Society (ACS) , Global Cancer Statistics 2024 Report, 2024, American Cancer Society, https://pressroom.cancer.org/GlobalCancerStatistics2024.

[3] Mann R. M. , Athanasiou A. , Baltzer P. A. , Camps-Herrero J. , Clauser P. , Fallenberg E. M. , Forrai G. , Fuchsjäger M. H. , Helbich T. H. , Killburn-Toppin F. , and Lesaru M. , Breast Cancer Screening in Women With Extremely Dense Breasts: Recommendations of the European Society of Breast Imaging (EUSOBI), European Radiology. (2022) 32, no. 6, 4036–4045, 10.1007/s00330-022-08617-6. 35258677 PMC9122856

[4] Ucar H. , Kaçar E. , and Karaca R. , The Contribution of a Solid Breast Mass Gray-Scale Histographic Analysis in Ascertaining a Benign-Malignant Differentiation, Journal of Diagnostic Medical Sonography. (2022) 38, no. 4, 317–322, 10.1177/87564793221078205.

[5] Han J. , Li F. , Peng C. , Huang Y. , Lin Q. , Liu Y. , Cao L. , and Zhou J. , Reducing Unnecessary Biopsy of Breast Lesions: Preliminary Results With Combination of Strain and Shear-Wave Elastography, Ultrasound in Medicine & Biology. (2019) 45, no. 9, 2317–2327, 10.1016/j.ultrasmedbio.2019.05.015, 2-s2.0-85067180854. 31221510

[6] Zhou D. , Bousquet O. , Lal T. N. , Weston J. , and Schölkopf B. , Learning With Local and Global Consistency, Advances in Neural Information Processing Systems. (2004) 16, 321–328.

[7] Wang Q. , Lin J. , and Yuan Y. , Salient Band Selection for Hyperspectral Image Classification via Manifold Ranking, IEEE Transactions on Geoscience and Remote Sensing. (2017) 55, no. 12, 6689–6700, 10.1109/TGRS.2017.2742986. 27008675

[8] Vizcarra J. , Place R. , Tong L. , Gutman D. , and Wang M. D. , Fusion in Breast Cancer Histology Classification, In Proceedings of the 10th ACM International Conference on Bioinformatics, Computational Biology and Health Informatics. (2019) 485–493, 10.1145/3307339.3342168, 2-s2.0-85073156989. PMC7339913 32637941

[9] Snigdha V. and Nair L. S. , Hybrid Feature-Based Invasive Ductal Carcinoma Classification in Breast Histopathology Images, Machine Learning and Autonomous Systems: Proceedings of ICMLAS 2021, 2021, Springer Nature Singapore, 515–525, 10.1007/978-981-16-8268-1_50.

[10] Roy S. D. , Das S. , Kar D. , Schwenker F. , and Sarkar R. , Computer-Aided Breast Cancer Detection Using Ensembling of Texture and Statistical Image Features, Sensors. (2021) 21, no. 11, 10.3390/s21113628, 34071029. PMC8197148 34071029

[11] Hirra M. , Usman F. , Qamar R. A. , Naqvi M. A. , and Rizwan M. , Breast Cancer Classification From Histopathological Images Using Patch-Based Deep Learning Modeling, IEEE Access. (2021) 9, 24273–24287, 10.1109/ACCESS.2021.3056516.

[12] Jiang Y. Z. , Liu Y. , Xiao Y. , Hu X. , Jiang L. , Zuo W. J. , Ma D. , Ding J. , Zhu X. , Zou J. , Verschraegen C. , Stover D. G. , Kaklamani V. , Wang Z. H. , and Shao Z. M. , Molecular Subtyping and Genomic Profiling Expand Precision Medicine in Refractory Metastatic Triple-Negative Breast Cancer: The FUTURE Trial, Cell Research. (2021) 31, no. 2, 178–186, 10.1038/s41422-020-0375-9, 32719455. 32719455 PMC8027015

[13] Yang C. , Liu H. , Feng X. , Shi H. , Jiang Y. , Li J. , and Tan J. , Research Hotspots and Frontiers of Neoadjuvant Therapy in Triple-Negative Breast Cancer: A Bibliometric Analysis of Publications Between 2002 and 2023, International Journal of Surgery. (2024) 110, no. 8, 4976–4992, 10.1097/JS9.0000000000001586, 39143709. 39143709 PMC11326012

[14] Shaila S. G. , Gurudas V. R. , and Monish L. , Breast Cancer Detection Based on Deep Neural Network Using Multi-Model Features, Proceedings of the 2022 International Conference on Artificial Intelligence and Data Engineering (AIDE), 2022, IEEE, 884–888, 10.1109/IC3IOT53935.2022.9767995.

[15] Zhou X. , Wu X. , Wang L. , Guo J. , Wu Q. , Song W. , Zhao Y. , Feng Z. , Wu S. , Zhang L. , and Gong X. , Metaplastic Breast Carcinoma: A Retrospective Study of 26 Cases, International Journal of Clinical and Experimental Pathology. (2021) 14, no. 3, 355–362, 33786152. 33786152 PMC7994148

[16] Park M. , Kim D. , Ko S. , Kim A. , Mo K. , and Yoon H. , Breast Cancer Metastasis: Mechanisms and Therapeutic Implications, International Journal of Molecular Sciences. (2022) 23, no. 12, 10.3390/ijms23126806, 35743249. PMC9224686 35743249

[17] Gurudas V. R. , Shaila S. G. , and Vadivel A. , Breast Cancer Detection and Classification From Mammogram Images Using Multi-Model Shape Features, SN Computer Science. (2022) 3, no. 5, 10.1007/s42979-022-01290-y.

[18] Awan R. and Rajpoot N. , Deep Autoencoder Features for Registration of Histology Images, Annual Conference on Medical Image Understanding and Analysis, 2018, Springer, 371–378.

[19] Sanyal R. , Kar D. , and Sarkar R. , Carcinoma Type Classification From High-Resolution Breast Microscopy Images Using a Hybrid Ensemble of Deep Convolutional Features and Gradient Boosting Trees Classifiers, IEEE/ACM Transactions on Computational Biology and Bioinformatics. (2022) 19, no. 4, 2124–2136, 10.1109/TCBB.2021.3055102. 33819160

[20] Bagchi P. P. and Sarkar R. , A Multi-Stage Approach to Breast Cancer Classification Using Histopathology Images, Diagnostics. (2023) 13, no. 1, 10.3390/diagnostics13010126, 36611418. PMC9818545 36611418

[21] Mohammed A. , Amer E. , Noor Eldin S. , Khaled J. , Hossam M. , Elmasry N. , and Adnan G. , The Impact of Data processing and Ensemble on Breast Cancer Detection Using Deep Learning, Journal of Computing and Communication. (2022) 1, no. 1, 27–37, 10.21608/jocc.2022.218453.

[22] Deepa B. G. , Senthil S. , Gupta Rahil M. , and Shah Vishakha R. , Augmentation of Classifier Accuracy Through Implication of Feature Selection for Breast Cancer Prediction, International Journal of Recent Technology and Engineering. (2019) 8, no. 2, 6396–6399.

[23] Asha S. B. , Gopakumar G. , and Subrahmanyam G. R. K. S. , Saliency and Ballness Driven Deep Learning Framework for Cell Segmentation in Bright Field Microscopic Images, Engineering Applications of Artificial Intelligence. (2023) 118, 105704, 10.1016/j.engappai.2022.105704.

[24] Sharmin S. , Ahammad T. , Talukder M. A. , and Ghose P. , A Hybrid Dependable Deep Feature Extraction and Ensemble-Based Machine Learning Approach for Breast Cancer Detection, IEEE Access. (2023) 11, 87694–87708, 10.1109/ACCESS.2023.3288493.

[25] Venugopal V. S. and Nair J. J. , Tuba M. , Akashe S. , and Joshi A. , Ensemble Deep Learning Model for Breast Histopathology Image Classification, ICT Infrastructure and Computing, 2023, Springer, 499–509, 10.1007/978-981-19-5331-6_51.

[26] Patel R. , Klein P. , Tiersten A. , and Sparano J. A. , An Emerging Generation of Endocrine Therapies in Breast Cancer: A Clinical Perspective, NPJ Breast Cancer. (2023) 9, no. 1, 10.1038/s41523-023-00523-4, 37019913. PMC10076370 37019913

[27] Shaila S. G. , Bhat G. , Gurudas V. R. , Suresh A. , and Hithyshi K. , Satapathy S. C. , Bhateja M. , and Balas V. E. , BRCA1 Genomic Sequence-Based Early Stage Breast Cancer Detection, Proceedings of International Conference on Recent Trends in Computing. Lecture Notes in Networks and Systems, 2023, Springer, 249–257.

[28] Shaila S. G. , Inamdar V. , Bhat R. , Hithyshi K. , and Suresh A. , Early Detection of Breast Cancer Based on HER-2 DNA Genomic Sequence, Proceedings of the International Conference on Applications of Machine Intelligence and Data Analytics (ICAMIDA 2022), 2023, Atlantis Press, 448–455, 10.2991/978-94-6463-136-4_38.

[29] Gurudas V. R. , Shaila S. G. , and Vadivel A. , Morphological and Textural Data Fusion for Breast Cancer Classification Based on Inter and Intra Group Variances, International Journal of Intelligent Engineering & Systems. (2024) 17, no. 3, 690–700, 10.22266/ijies2024.0430.56.

[30] Oliveira M. , Rugo H. S. , Howell S. J. , Dalenc F. , Cortes J. , Gomez H. L. , Hu X. , Toi M. , Jhaveri K. , Krivorotko P. , Loibl S. , Morales Murillo S. , Okera M. , Nowecki Z. , Park Y. H. , Sohn J. H. , Tokunaga E. , Yousef S. , Zhukova L. , Fulford M. , Andrews H. , Wadsworth I. , D′Cruz C. , Turner N. C. , and CAPItello-291 study group , Capivasertib and Fulvestrant for Patients With Hormone Receptor-Positive, HER2-Negative Advanced Breast Cancer (CAPItello-291): Patient-Reported Outcomes From a Phase 3, Randomised, Double-Blind, Placebo-Controlled Trial, Lancet Oncology. (2024) 25, no. 9, 1231–1244, 10.1016/S1470-2045(24)00373-5, 39214106. 39214106

[31] Cao H. , Wang Y. , Chen J. , Jiang D. , Zhang X. , Tian Q. , and Wang M. , Swin-Unet: Unet-Like Pure Transformer for Medical Image Segmentation, European Conference on Computer Vision, 2022, Springer Nature Switzerland, 205–218.

[32] Zhang Y. , Zhang X. , Li J. , Xu W. , and Chen L. , Cross-scale multi-instance learning for Pathological Image Diagnosis, Medical Image Analysis. (2024) 94, 103124, 10.1016/j.media.2024.103124, 38428271. 38428271 PMC11016375

[33] Lu M. Y. , Chen J. , Wang D. K. , Singh R. , and Mahmood F. , A Multimodal Generative AI Copilot for Human Pathology, Nature. (2024) 634, no. 8033, 466–473, 10.1038/s41586-024-07618-3, 38866050. 38866050 PMC11464372

[34] McGenity C. , Clarke E. L. , Jennings C. , Matthews G. , Freduah-Agyemang H. , and Stocken D. D. , Artificial Intelligence in Digital Pathology: A Systematic Review and Meta-Analysis of Diagnostic Test Accuracy, NPJ Digital Medicine. (2024) 7, no. 1, 10.1038/s41746-024-01106-8, 38704465. PMC11069583 38704465

[35] Al Nemer A. M. , Application of Artificial Intelligence in the Field of Breast Pathology Diagnosis: Narrative Review, Journal of Medical Artificial Intelligence. (2024) 7, no. 2, 1–9, 10.21037/jmai-24-22.

[36] Shen I. Z. and Zhang L. , Digital and Artificial Intelligence-Based Pathology: Not for Every Laboratory - A Mini-review on the Benefits and Pitfalls of Its Implementation, Journal of Clinical and Translational Pathology. (2025) 5, no. 2, 79–85, 10.14218/jctp.2025.00007, 40823629. 40823629 PMC12356165

[37] Datwani S. , Kaur A. G. , and Patel M. , Artificial Intelligence in Breast Pathology: Overview and Future Directions, Medical Image Analysis. (2025) 98, 103510, 10.1016/j.media.2025.103510.

[38] Wang K. , Zheng F. , Cheng L. , Dai H.-N. , Dou Q. , and Qin J. , Breast Cancer Classification From Digital Pathology Images via Connectivity-Aware Graph Transformer, IEEE Transactions on Medical Imaging. (2024) 43, no. 8, 2854–2865, 10.1109/TMI.2024.3381239, 38526888. 38526888

[39] Cheng J. C. , Wang Y. , Li R. , and Zhao P. , Large-Scale Transformer Models for Histopathological Image Segmentation: A Benchmarking Study, Nature Biomedical Engineering. (2024) 9, 1021–1034, 10.1038/s41551-024-01245-8.

[40] Liu X. , Chen D. , Huang Y. , and Xu T. , Foundation Models in Computational Pathology: Current Progress and Future Challenges, Annual Review of Biomedical Data Science. (2025) 8, 225–248, 10.1146/annurev-biodatasci-103123-s095814.

[41] Aresta G. , Araújo T. , Kwok S. , Chennamsetty S. S. , Safwan M. , Alex V. , Marami B. , Prastawa M. , Chan M. , Donovan M. , Fernandez G. , Zeineh J. , Kohl M. , Walz C. , Ludwig F. , Braunewell S. , Baust M. , Vu Q. D. , To M. N. N. , and Aguiar P. , BACH: Grand Challenge on Breast Cancer Histology Images, Medical Image Analysis. (2019) 56, 122–139, 10.1016/j.media.2019.05.010, 2-s2.0-85067343074, 31226662. 31226662

[42] Spanhol F. A. , Oliveira L. S. , Petitjean C. , and Heutte L. , A Dataset for Breast Cancer Histopathological Image Classification, IEEE Transactions on Biomedical Engineering. (2016) 63, no. 7, 1455–1462, 10.1109/TBME.2015.2496264, 2-s2.0-84978091692. 26540668

[43] BreaKHis Dataset,” [Online]. https://data.mendeley.com/datasets/jxwvdwhpc2/1.

